# Unveiling the promising anticancer effect of copper-based compounds: a comprehensive review

**DOI:** 10.1007/s00432-024-05641-5

**Published:** 2024-04-25

**Authors:** Sara Abdolmaleki, Alireza Aliabadi, Samad Khaksar

**Affiliations:** 1 Department of Pharmaceutical Chemistry, School of Science and Technology, The University of Georgia, Tbilisi, Georgia; 2 Pharmaceutical Sciences Research Center, Health Institute, School of Pharmacy, Kermanshah University of Medical Sciences, Kermanshah, Iran

**Keywords:** Copper-based compounds, Copper nanoparticles, Anticancer activity, Apoptosis, Angiogenesis

## Abstract

Copper is a necessary micronutrient for maintaining the well-being of the human body. The biological activity of organic ligands, especially their anticancer activity, is often enhanced when they coordinate with copper(I) and (II) ions. Copper and its compounds are capable of inducing tumor cell death through various mechanisms of action, including activation of apoptosis signaling pathways by reactive oxygen species (ROS), inhibition of angiogenesis, induction of cuproptosis, and paraptosis. Some of the copper complexes are currently being evaluated in clinical trials for their ability to map tumor hypoxia in various cancers, including locally advanced rectal cancer and bulky tumors. Several studies have shown that copper nanoparticles can be used as effective agents in chemodynamic therapy, phototherapy, hyperthermia, and immunotherapy. Despite the promising anticancer activity of copper-based compounds, their use in clinical trials is subject to certain limitations. Elevated copper concentrations may promote tumor growth, angiogenesis, and metastasis by affecting cellular processes.

## Introduction

Metal-based compounds offer a promising opportunity to develop new cancer treatments (Aliabadi et al. 2021a, b; Abdolmaleki et al. 2021a, b). These compounds with their unique properties and mechanisms of action have shown many advantages in the fight against cancer compared to other chemical compounds (Abdolmaleki et al. 2023a,b, 2022b; Adibi et al. 2019). One of the advantages of these compounds is their ability to target specific cellular processes or proteins involved in cancer cell growth and survival (Khaksar et al. 2023a,b; Abdolmaleki et al. 2021a, b). The targeted approach can potentially minimize damage to healthy cells and reduce side effects compared to conventional chemotherapeutic agents (Abdolmaleki et al. 2020; Heydari et al. 2020a, b; Khaksar et al. 2023a, b). Metal ions commonly used in this method include platinum, ruthenium, gold, and copper (Abdolmaleki et al. 2023a, b).

Studies have shown that the complexation of copper ions with some organic ligands increases the biological activity of the resulting compound (Abdolmaleki et al. 2023a, b; Abdolmaleki et al. 2016; Abdolmaleki and Ghadermazi 2017). In 2023, our group reported significant cytotoxicity of copper(II) complexes with pyridine-2,6-dicarboxylate against BEL-7404 cells. The mechanistic study of the anticancer effect of this complex showed that it generates high ROS and stops the progression of the cell cycle in the G_2_/M phase. Treatment of BEL-7404 cells with copper(II) complex induced apoptosis through a caspase-dependent mitochondrial signaling pathway (Abdolmaleki et al. 2023a, b). In our other study, a strong cytotoxic effect was also observed for two mixed-ligand mono- and binuclear copper(II) complexes containing pyridine-2,6-dicarboxylate derivatives and 2-aminopyrimidine. In this study, the complexes were found to have stronger inhibitory effects than their ligands in all three cell lines tested, including βTC, MCF7, and HT29. The strongest antiproliferative properties of these complexes were observed in the MCF7 cell line (Abdolmaleki et al. 2016). This effect was also confirmed in our study on a polymeric copper(II) complex synthesized from pyridine dicarboxamide derivatives. Similar to a previous study, cancer cells showed the highest sensitivity to the copper(II) complex compared to the tested ligands and oxaliplatin (Abdolmaleki et al. 2017). In 2023, Climova et al. reported a remarkable inhibitory effect of three copper(II) complexes formed from hydrazonamide derivatives on cancer cell lines. They found that among the compounds tested, the complex containing N′-(benzylidene)-6-chloropyrazine-2-carbohydrazonamide was more effective and selective against LN229 cancer cells (Climova et al. 2023). In another study, copper(I) complexes with tridentate homoscorpionate tris(pyrazolyl)borate and monodentate phosphine auxiliary ligands were investigated due to their capacity to suppress the proliferation of cancer and normal cells. Some of them were found to be selective against tumors by inhibiting 26S proteasome activity associated with endoplasmic reticulum (ER) stress and activation of unfolded protein response (UPR). In this case, no signs of biochemical features related to apoptosis were observed, and morphological findings showed significant cytoplasmic vacuolization associated with a cell death process similar to paraptosis (Gandin et al. 2014).

Studies have shown that copper-based compounds act via a mechanism directed with DNA. The antitumor activities of these compounds rely on the interaction between copper and the ligand. The toxicity of copper arises from its ability to undergo cycles of oxidation and reduction, to remove ions from the binding sites of enzymes, bind strongly to DNA and cause DNA breaks (Marzano et al. 2009). Modification of copper-based compounds through the ligand scaffold increases its affinity, specificity, and stability toward DNA (Ceramella et al. 2020). These compounds can bind to DNA through noncovalent interactions, such as binding to major or minor grooves, intercalation, or electrostatic binding. Certain copper-based compounds produce ROS that overwhelm the body's antioxidant defenses and lead to oxidative damage in the cytoplasm, mitochondria, and DNA (Molinaro et al. 2020). A significant group of copper-based compounds acts as inhibitors of topoisomerases (Top) 1 and 2, leading to significant DNA damage, cell cycle arrest, and ultimately cell death (Shobha Devi et al. 2018). These compounds convert transient DNA enzyme complexes into lethal DNA breaks (Thomas and Pommier 2019).

Although copper is an essential trace element in cells, its high reactivity makes it toxic, which is why the body strictly regulates its levels. Copper metabolism is impaired in neoplastic diseases, and elevated blood copper levels are related to the progression and recurrence of various cancers (Geraki et al. 2002). The exact molecular mechanism behind the link between elevated copper and malignant cells is not yet fully understood, but it is suspected that copper has a role in the angiogenesis of early-stage tumors. According to studies, copper stimulates the growth and movement of endothelial cells, which are involved in the formation of blood vessels, and acts as a cofactor for angiogenic factors. The human copper transporter (hCTR1) also affects cell signaling pathways in embryogenic cells and may contribute to cancer development (Narayanan et al. 2013). The differential response of tumor cells and normal cells to copper suggests that copper-based compounds may be developed as anticancer agents (Wee et al. 2013; Khan et al. 2016). This depends on the choice of organic motifs, scaffolds, and the amount of donor atoms (Evans et al. 2015). In general, ligands have a prominent role in the cytotoxicity of metal compounds in cancer research (Abdolmaleki et al. 2018, 2017, 2019; Heydari et al. 2020a, b; Abdolmaleki et al. 2022a, b; Aliabadi et al. 2022; Zohrevandi et al. 2022). In a study, Hussain et al. evaluated the potential of copper complexes with a biocompatible Schiff base ligand as promising options for next-generation anticancer drugs and NSAIDs. These complexes showed strong binding ability to a model protein, indicating their potential for targeted therapy. In in vitro studies, the complex with 1,10-phenanthroline (phen) as a ligand was found to have remarkable efficacy against MCF-7 breast cancer cells compared to two other complexes. The mechanism by which these complexes exerted their cytotoxic effects was further investigated, and ROS generation was found to play an important role. The observed morphological changes in the cancer cells indicated that late apoptosis was induced by treatment with the complexes. Animal studies conducted in vivo showed that some of these complexes exhibited dose-dependent anti-inflammatory and analgesic activities, suggesting their potential as NSAIDs. Computational studies supported the notion that these complexes interact with a COX2 inhibitor, confirming their potential mechanism of action as NSAIDs (Hussain et al. 2019).

Researchers have also explored the utilization of copper-containing nanoparticles in the treatment of cancer, which is very promising. These innovative compounds have the potential to transform cancer therapy through innovation, enabling more effective and targeted treatments with fewer side effects. The results show that the copper ions released by the nanoparticles can trigger oxidative stress and induce programmed cell death in cancer cells. Copper nanoparticles also can selectively attack cancer cells and spare healthy cells. This selectivity is attributed to the EPR effect, which enables nanoparticles to accumulate in tumor tissue by exploiting leaky blood vessels (Mariani et al. 2021; Aishajiang et al. 2023).

While many copper complexes have shown significant cytotoxicity in in vitro studies, few have been tested in animal models (Khan et al. 2016). Further research is needed to understand their mechanisms of action, optimize their therapeutic potential, and evaluate safety and efficacy in clinical trials, as the history of copper complexes in cancer therapy is relatively limited compared to other metal complexes such as platinum. Therefore, in this review article, we focus on the anticancer effect investigation of copper-based compounds, their mechanisms of action, and their use in clinical trials. Nanoparticles containing copper are discussed as effective agents for the diagnosis and treatment of cancer. The limitations and challenges of using copper-based compounds are also explained. Although the use of copper-based compounds in the treatment of cancer is still at an early stage of development, studies have shown promising results concerning their antiproliferative and antiangiogenic effects.

## Copper's chemical and biochemical properties

Copper is a vital nutrient for both plants and animals and serves as a cofactor in enzymes and as a component of pigments. In mammals, it is found mainly in the bloodstream and various organs (Elkanzi et al. 2023; Al-Fakeh et al. 2023). In humans, high copper concentrations can be harmful to cells, leading to the oxidation of DNA and structural changes in proteins and biomembranes. Copper ions, especially in blue copper proteins, play a role in redox reactions and electron transfer processes. Transition metal ions, including copper, exhibit selectivity in molecular recognition and can change their valence in redox reactions (Feng et al. 2023). One of the most important explanations for the cellular toxicity caused by copper is the ability of free copper ions to generate ROS.

Both copper(II) and (I) ions undergo oxidation and reduction reactions. When exposed to superoxide (O^2−^), ascorbic acid, or glutathione (GSH), copper(II) is reduced to copper(I). Copper(I) then can catalyze the production of hydroxyl radicals (^**.**^OH) from hydrogen peroxide (H_2_O_2_) through the Haber–Weiss reaction (Piroš et al. 2023). The highly reactive^**.**^OH interacts with biological molecules and causes damage by cleaving hydrogen from carbon atoms in amine-containing compounds and unsaturated fatty acids. This leads to the formation of protein and lipid radicals that contribute to oxidative damage in cells. Copper has been found to induce DNA damage and oxidation through the formation of ROS. GSH inhibits the formation of free radicals by copper ions in the presence of H_2_O_2_, ascorbate, and DNA. This defensive impact is due to the ability of GSH to stabilize copper(I) and thus prevent the creation of free radicals by redox cycling. Copper(II) also forms thioxyl radicals, copper cysteine, and copper methionine ions, possibly leading to the formation of metallothioneins (MT) and disulfides (RSSR), which can be harmful (Han 2023).

However, it is crucial to mention that the presence of free copper ions in cells is usually limited to less than one ion per cell. This suggests that the cells have a considerable capacity to chelate or bind copper, ensuring that it does not accumulate to toxic levels (Franco et al. 2023). Regulation of copper levels in the body is critical to maintaining health, as both excess and deficiency of this metal can be harmful. Metal-binding sequences, which are specialized domains rich in cysteine, methionine, or histidine, play a prominent role in maintaining the concentration of free copper in cells at an extremely low level of below 10–18 M (Bisaglia and Bubacco 2020).

ATP7A, also known as the Menkes protein, facilitates the absorption of copper from food in the stomach and small intestine. In normal human serum, most copper is bound to ceruloplasmin, an enzyme that contains 6 copper atoms in both the copper(II) and (I) states. However, this state of copper is not easily interchangeable. Copper in its interchangeable state is tightly attached to albumin and amino acids (Mhaske et al. 2020). The copper–histidine complex has been recognized as the primary copper–amino acid complex in human serum and human albumin has been discovered to create a ternary complex with copper-histidine (Ha et al. 2023; Song et al. 2022). During copper uptake, copper(II) is reduced to copper(I) and taken up by cells via transmembrane transporters. The major copper influx transporter in human cells is hCTR1, which is found predominantly in the plasma membrane and is expressed at higher levels in the liver, kidney, and heart. hCTR1 probably binds copper through its amino-terminal domain and transports it across the cell membrane (Magrì et al. 2022; Orlov et al. 2023).

Once in the cytoplasm, copper can form complexes with various ligands, with the majority being bound to GSH as copper(I) (Falcone et al. 2023). The Cu(I)-GS complex can then be transferred to intracellular proteins such as MT, which is important for metal detoxification (Ritacca et al. 2023). To prevent an accumulation of copper in mammalian cells, there are two closely related P-type ATPases known as ATP7A and ATP7B. These ATPases are responsible for facilitating the removal of copper from cells. ATP7A is found in various tissues other than the liver, while ATP7B is mainly found in the liver, kidney and to a lesser extent in the brain (Karpenko et al. 2023; Guan et al. 2023). Both proteins have a high affinity for copper and undergo regulated movement within cells when copper binds to them. Results have shown that copper transporters, including hCtr1, ATP7A, and ATP7B, play a role in importing, distributing, and exporting platinum-containing drugs such as cisplatin. This suggests that these copper transporters may influence the sensitivity of cancer cells to platinum-based medications. The mechanisms by which these transporters facilitate the transport of platinum drugs have not been fully elucidated (Zhao et al. 2023). However, their ability to discriminate between different metal ions suggests that changes in the expression of copper transporters may influence the cellular response to platinum drugs through secondary effects on other metabolic pathways, such as MT and GSH levels (Bisaglia and Bubacco 2020; Zhao et al. 2023).

## Overview of copper-based compounds

### Anticancer activity

The first discovery of the antitumor effect of copper-based compounds dates back to the 1960s (Graur et al. 2023; Jenkins et al. 2023). Since then, numerous papers have been devoted to investigating the chemical and biological properties of these complexes. Despite extensive research into the production of different types of copper-based compounds, there is little information on their physiological processing. Research has shown that the chelation of organic ligands with copper ions is important in enhancing their biological activity (Zhao et al. 2023).

Accordingly, copper(II) complexes of thiosemicarbazones (TSCs) (1a and b) were shown to have excellent inhibitory activity toward HeLa, HepG2, SGC-7901, and L02 cell lines compared with their corresponding ligands (Ma et al. 2014; Shao et al. 2014). In particular, these complexes showed promising results by inducing DNA fragmentation and increasing ROS production. Complex (1a) showed remarkable cytotoxicity against HeLa cells. This compound promoted apoptosis in the aforementioned cells via an oxidative DNA damage pathway, making it a potential anticancer agent. It was found that (1a) induces apoptosis in HeLa cells more strongly than (1b). The direct effects of (1a) on DNA replication, transcription, and protein synthesis are due to the DNA damage it causes. In general, copper complexes have shown promising anticancer activity in many reports, as they accumulate in tumors and selectively penetrate the membranes of cancer cells (Deegan et al. 2006; Deegan et al. 2007b, a). In particular, copper complexes containing N-donor ligands, such as TSCs, are effective against various cancer cell lines. In a study, the copper(II) complex containing 2-formylpyridine TSC (2) was reported to be able to inhibit ribonucleic acid (RNA)-dependent DNA polymerases as well as the transforming ability of Rous sarcoma virus (Kaska et al. 1978).

Ferrari et al. performed a study in which they modified derivatives of TSCs by replacing the 2-pyridine group with salicylaldehyde (Ferrari et al. 1999), pyridoxal (Ferrari et al. 2002), and 5-formyluracil (Ferrari et al. 1998). Among these derivatives, the salicylaldehyde TSC (H_2_ salt) showed selectivity for copper(II) and formed a dimeric metal complex (3). When in vitro evaluations were done with human leukemic U937 cells to study the effects of complex (3) on the suppression of cell proliferation and triggering of programmed cell death, the evaluations showed that this complex inhibited about 40% of cell proliferation, but no DNA fragmentation or apoptosis was confirmed. This behavior can be attributed to the specific arrangement of the copper atoms in a square-planar polyhedron of complex (3). In contrast, (4) formed with the pyridoxal-TSC ligand (Ferrari et al. 1994) and the compounds (5–7) formed with the 5-formyl-uracil-TSC ligand all exhibited a five-coordinated structure and induced process of breaking DNA molecules into smaller fragments that ultimately led to apoptosis [65]. Investigation of the cytotoxicity of the copper(II) complexes of isatin TSCs, particularly (8) and (9), on the U937 cell line revealed that both copper(II) complexes could strongly inhibit cell proliferation, with cytotoxicity of 70% at a dose of 20 μg mL^−1^ (Rodriguez-Arguelles et al. 1999). In another study, copper(II) complexes of 6-nitropiperonal TSC (10a-d) were evaluated for their interaction with DNA and HSA. These complexes exhibited antioxidant properties and strong binding to DNA, with a binding constant of 10^5^ M^−1^. Binding to DNA was significantly stronger than binding to HSA. Here, the complexes were reported to have a similar core structure to oxolinic acid, a type of antibiotic known as quinolone that acts specifically on DNA gyrase and Top (IV). The strength of its interaction with DNA gyrase B varied between dissociation constants of 0.37 to 1.27 µM, while its binding to Top (IV) was even more potent with dissociation constants of 4.32 to 24.65 µM (Beckford and Webb 2017).

**Fig. 1:**
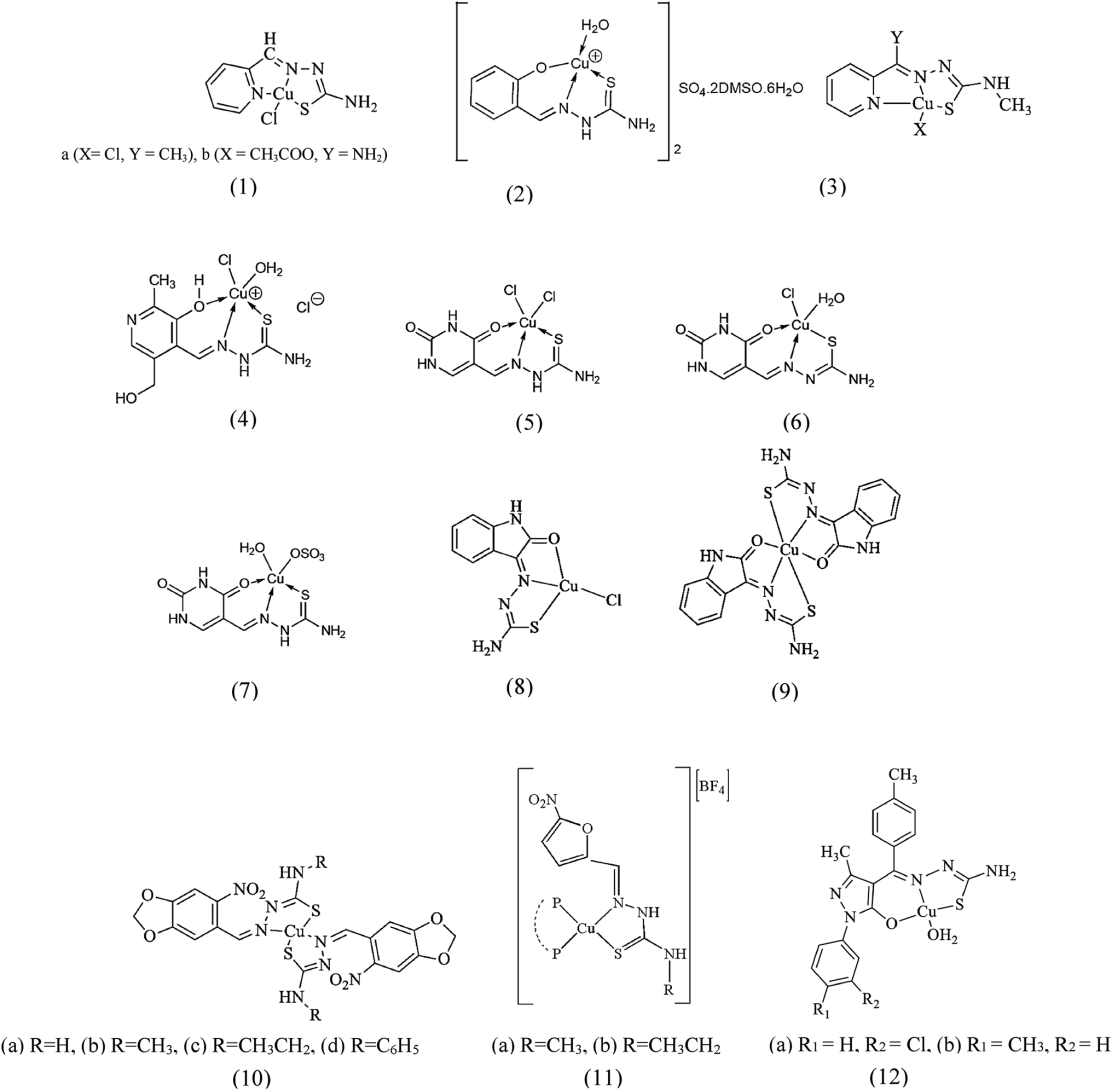
Structure of copper(II) complexes containing thiosemicarbazones

In 2023, Machado et al. demonstrated that copper(I) complexes (11a and b) containing a bioactive TSC ligand and a phosphane ligand (PP) exhibited dual anticancer and antiparasitic properties. Here, the in vitro anti-trypanosome and anticancer activities of the complexes against *Trypanosoma cruzi* and the cancer cells OVCAR3 and PC3 were investigated. To determine their selectivity against parasites and cancer cells, cytotoxicity against normal monkey kidney cells (VERO) and human skin fibroblasts (HDF) was also investigated. The synthesized heteroleptic complexes showed higher cytotoxicity against *T. cruzi* and chemoresistant prostate PC3 cells compared with the benchmark drugs nifurtimox and cisplatin. These complexes were observed to be effectively taken up by OVCAR3 cells, especially those containing the dppe-PP ligand, and activated the cell death mechanism mediated by apoptosis. However, there was no clear evidence of ROS production induced by these complexes (Machado et al. 2023). In another study, the affinity for binding of copper(II) complexes containing TSC (12a and b) with pET30a plasmid DNA was evaluated. The results proved that both complexes had nuclease activity, with more efficient DNA cleavage observed when the complexes were mixed with H_2_O_2_. Binding to DNA occured by partial intercalation binding and DNA degradation depended on the concentration of the complex. Complete DNA cleavage was observed at a concentration of 50 μM. (12a) showed stronger binding affinity to DNA compared to 12b, as evidenced by a greater increase in viscosity (Baldini et al. 2004). The structure of the above copper(II) complexes is represented in Fig. 1.

According to the literature, the biological activities of Schiff base ligands, such as anticancer, antibacterial, and antiviral activities, are enhanced when they coordinate with metal ions, especially copper(I) and (II) ions (Gomathi and Andy 2013). In a study, strong cytotoxicity and remarkable electrochemical activity were observed for a dinuclear copper(II) complex (13) prepared from a Schiff base ligand of N-(2-hydroxylbenzylidene)-benzo[d]imidazol-2-amine. In addition, (13) showed binding to DNA by groove binding and electrostatic interaction, resulting in conformational changes in the DNA structure. This complex could effectively cleave the supercoiled plasmid DNA into nicked and linear forms by an oxidative mechanism. In cell experiments, (13) was found to readily penetrate cancer cells, including the nucleus, induce cell apoptosis and exhibit cytotoxicity similar to cisplatin in tests with the cell lines HeLa and A549. In addition, (13) showed a higher inhibitory effect against MCF-7 cell lines compared to other cell lines (Zhao et al. 2022). Zhong et al. reported that a mononuclear distorted octahedral copper(II) complex (14) prepared from the Schiff base ligand (Z)-2-hydroxy-N'-(2-oxoindolin-3-ylidene)benzohydrazide (Zhong et al. 2007) exhibited pronounced cytotoxicity in four different human cancer cell lines (SPCA-1, Tb, MGC, and K562), and its potency was even higher compared to similar compounds reported previously (Bestwick et al. 2005). The authors suggested that the enhanced potential anticancer activity of the compounds could be attributed to the production of toxic copper(I) species through enzymatic reduction processes within the cells (Kostova et al. 2005). In another study, Schiff base compounds developed by attaching pharmacophores, such as amino group-bearing TSCs, to the central ketone function of ketoprofen (Saha et al. 2001; Padhye et al. 2005) were evaluated for their anticancer properties. These compounds showed remarkable antiproliferative activity against breast cancer cells when reacted with transition metals such as copper. In addition, the authors synthesized a selective COX-2 inhibitor derivative of ketoprofen, compound (15), which showed prominent effects on the inhibition of cell growth and promote programmed cell death in COX-2-positive cells (Ahmed et al. 2007). In addition, the authors synthesized a selective COX-2 inhibitor derivative of ketoprofen, compound (15), which showed outstanding effects on inhibiting cell growth and promoting programmed cell death in COX-2-positive cells (Zhao et al. 2023; Ahmed et al. 2007). Adsule et al. prepared Schiff base copper complexes of quinoline-2-carboxaldehyde ligands and investigated their ability to cause cytotoxicity and induce apoptosis in prostate cancer cell lines. The compounds induced apoptosis in prostate cancer cell lines without triggering oxidative stress, and the addition of thiocarbonyl side chains increased their anticancer activity. Here, compound (16) showed potent proteasome inhibitory activity (Adsule et al. 2006). Cerchiaro et al. prepared isatin-Schiff base copper(II) complexes that showed keto-enol equilibria and high stability (Cerchiaro et al. 2005). These complexes triggered the activation of the apoptotic program in human promonocytes and neuroblastoma cells. Two specific complexes, (17) and (18), induced apoptosis via the mitochondrial pathway. There was a correlation between the amount of apoptosis and the uptake of copper within the cells. It was hypothesized that these complexes transport copper into cells, produce ROS, and act specifically on mitochondria due to their delocalized lipophilic cations (Filomeni et al. 2007).

In 2017, Kathiresan et al. pointed out that a group of four copper(II) complexes with mixed ligands of the general formula [Cu(L)(diimine)](ClO_4_) (19), where the diimines are phen, 2,2’-bipyridine (bpy), 4,4’-dimethyl-2,2’-bipyridyl (dmbpy), or 2,2’-dipyridylamine (dpa) were able to effectively cause single-strand breakage in the pUC18 plasmid DNA by introducing ascorbic acid. Moreover, bovine serum albumin (BSA) showed static quenching when interacting with these complexes. Their ability as free radical scavengers and anti-inflammatory agents was evaluated using* 2,2-diphenylpicrylhydrazyl* (DPPH) and protein denaturation techniques. Moreover, in vitro inhibitory effect studies against AGS cancer cells showed strong anticancer effects (Kathiresan et al. 2017).

**Fig. 2:**
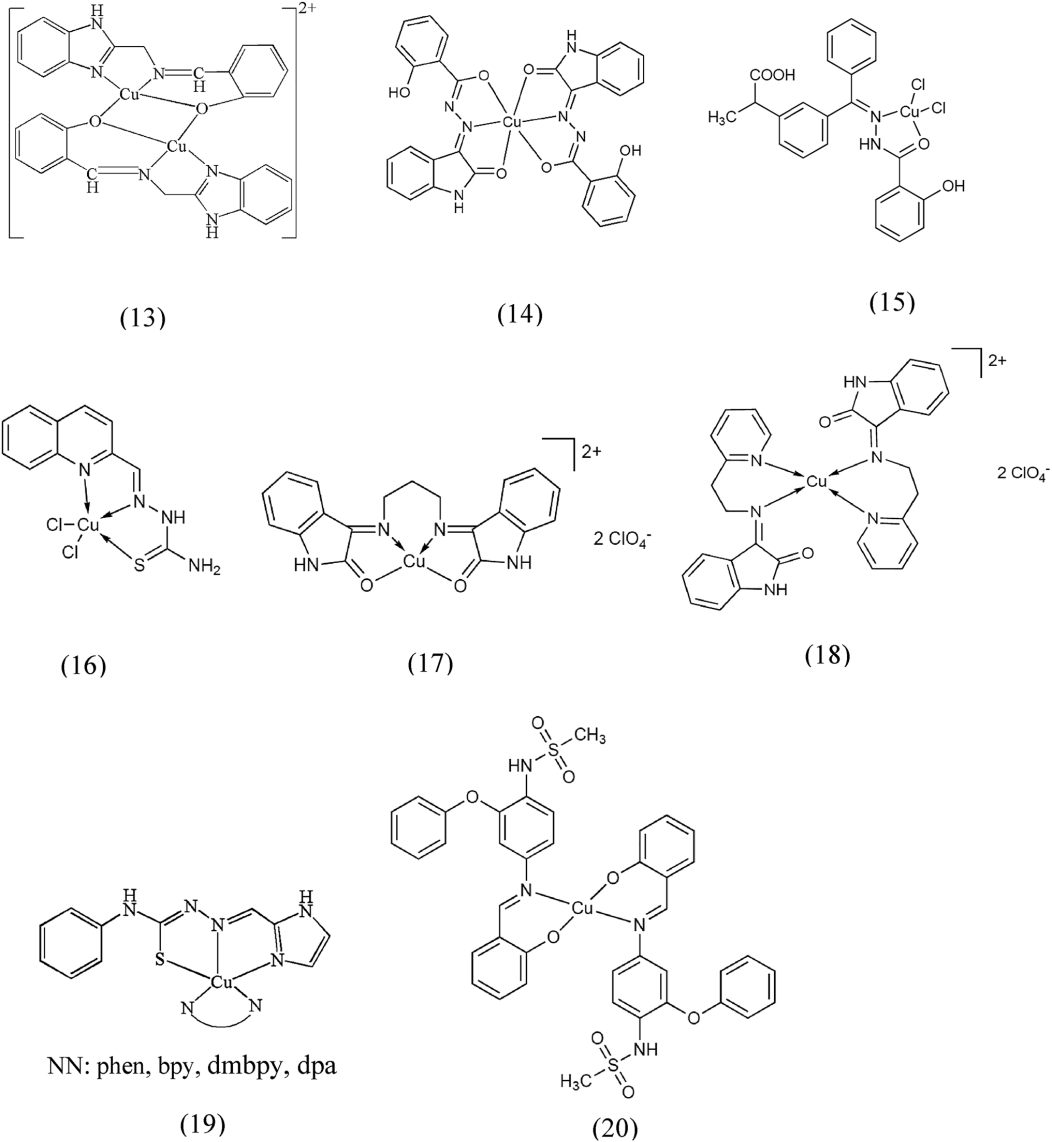
Structure of copper(II) complexes containing Schiff Base ligands

In another study, distorted square planar copper(II) complexes (20) synthesized as Schiff base derivatives of nimesulide were found to have a significant cytotoxic effect on both pancreatic tumor cell lines BxPC-3 (COX-2 positive) and MiaPaCa (COX-2 negative), with IC_50_ values of 3 to 26 μM for the COX-2-positive cell line and 5 to 9 μM for the COX-2-negative cell line, which was more remarkable compared with nimesulide with IC_50_ values of 35 μM for the COX-2-positive cell line and more than 100 μM for the COX-2-negative cell line. The observed biological activity of these compounds was attributed to the suppression of VEGF and COX-2 cells, as well as the down-regulation of the antiapoptotic proteins Bcl-2 and Bcl-XL (Ambike et al. 2007; Kroemer and Reed 2000). The structure of the aforementioned copper(II) complexes is illustrated in Fig. 2.

In a study, the copper(II) complex containing imidazole (21) was reported to have the strongest antitumor activity against the B16 mouse melanoma cell line (Tamura et al. 1987). In another study, a copper(II) complex (22) containing 1-methyl-4,5-diphenylimidazole with distorted octahedral geometry significantly delayed cell division, sister chromatid exchanges (SCEs), and mitotic indices (MIs) in cultured human lymphocytes (Raptopoulou et al. 1998). It also reduced proliferation rate indices (PRIs), indicating its cytostatic and cytotoxic effects. Low concentrations of compound (22) caused the unwinding of the plasmid pKS DNA and cleavages on both closed, supercoiled and open, relaxed forms of DNA, with guanosines being favored. In addition, strand cross-linking of DNA bases was shown in ds-DNA from calf thymus and plasmid DNA treated with the compound (22). According to studies, benzimidazole and its derivatives were recognized for their diverse biological properties, including antibacterial, antiviral, anticancer, and antifungal activities (Habib et al. 1997). For example, the trigonal bipyramidal copper(II) complex (23) five-coordinated with two chelating 2-(4-thiazolyl)benzimidazole or thiabendazole ligands and a chloride ion (Devereux et al. 2004) showed significant chemotherapeutic potential when tested against CAL-27 and SK-MEL-31 cells. In another study, Saczewski et al. synthesized and characterized a group of copper-based complexes with bidentate chelating ligands derived from benzimidazole (Saczewski et al. 2006). Among these compounds, complex (24) showed highly potent Cu,Zn-SOD activity in vitro (IC_50_ = 0.09 μM). This value was in line with Cu,Zn-SOD mimetics of ideal molecular weight reported in the literature, including heterodinuclear copper(II)–Zinc(II) complexes with imidazolate bridges. Moderate inhibitory effects on cell growth were observed in in vitro anticancer evaluations involving seven human tumor cell lines. Lung cancer cell line A427 showed the highest level of sensitivity to compound (24) (IC_50_ = 4.76 to 10.12 μM) (Szilagyi et al. 2005; Patel et al. 2002).

**Fig. 3:**
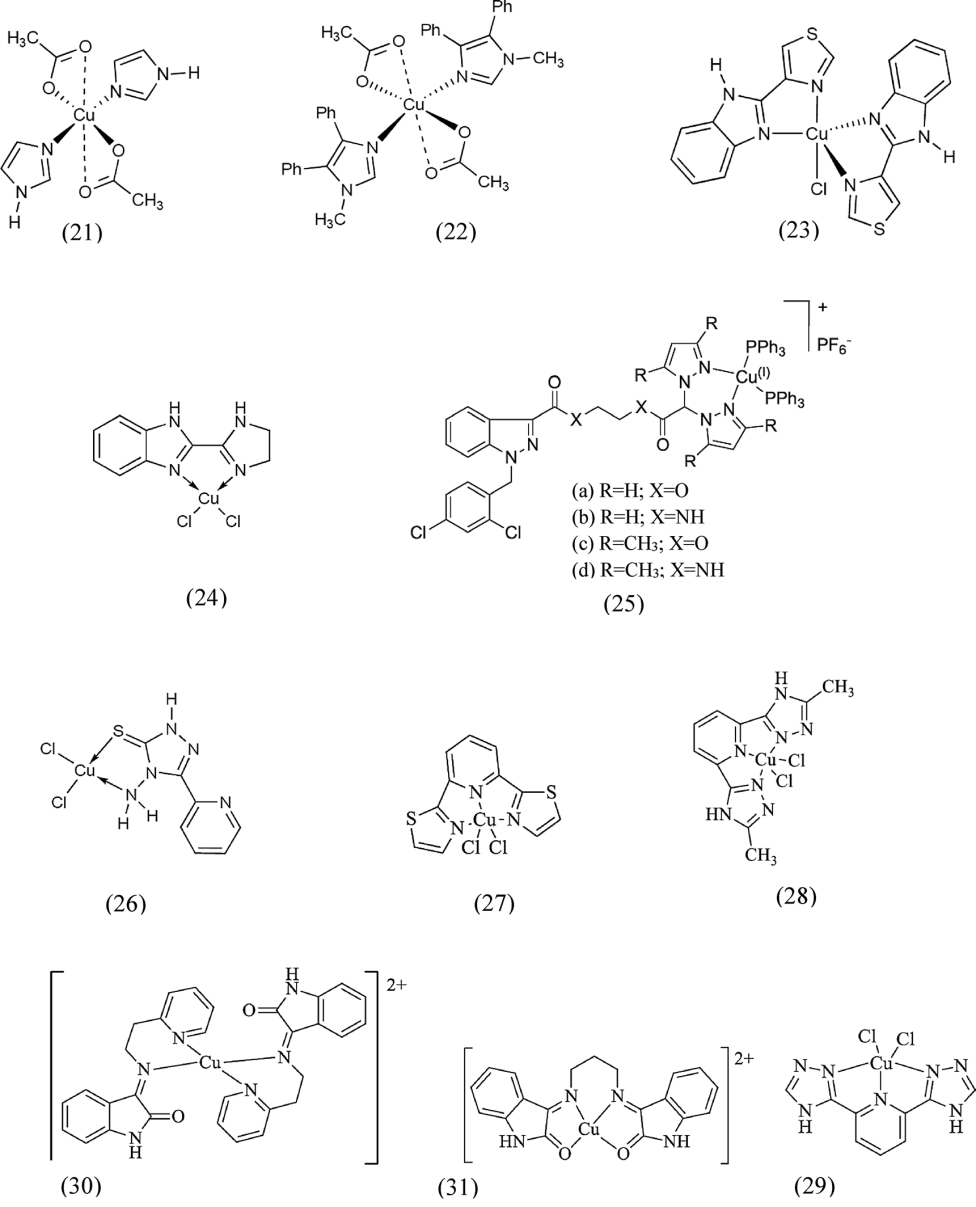
Structure of copper(II) complexes containing imidazole, benzimidazole, pyrazole, triazole, pyridine, and isatin diimine

In 2022, Bello et al. concluded that copper(I) and (II) complexes (25a-d) containing pyrazole derivatives exhibited significant antitumor activity and were stronger than the standard drug cisplatin in a variety of human tumor cells. In addition, these complexes were able to overcome resistance to oxaliplatin and multidrug resistance. Notably, compared with cisplatin, the complexes showed superior efficacy when tested against 3D spheroids of PSN-1 pancreatic cancer cells. Among these complexes, the copper(I) complex (25d) containing *triphenylphosphine* (PPh_3_) showed the most hopeful results. Mechanistic evaluations showed that (25d) induced cancer cell death by an alternative form of apoptosis (Del Bello et al. 2022). Dallavalle et al. (Dallavalle et al. 2002) found that the copper(II) complex containing triazole (26) exhibited cytotoxic activity comparable to cisplatin in human fibrosarcoma HT1080 cells. Interestingly, normal human fibroblasts were not affected by (26). Further studies revealed that (26) triggered a particular model of programmed cell death characterized by the formation of large cytoplasmic vacuoles without nuclear fragmentation or caspase-3 activation. This suggests that (26) induces a non-apoptotic form of programmed cell death by inhibiting caspase-3-dependent apoptotic signaling pathways (Tardito et al. 2006). Similar results were observed in human ovarian adenocarcinoma cells treated with certain copper(I) phosphine complexes (Marzano et al. 2008). Tridentate copper(II) complexes with pyridine ring (27–29) were shown cytotoxic effects against HeLa, HepG2, and BEL-7402 cell lines. Their IC_50_ values (0.93 × 10^–4^–2.72 × 10^–4^ M) were comparable to cisplatin (0.14 × 10^–4^–0.26 × 10^–4^ M) and lower than 5-fluorouracil (8.76 × 10^–4^–24.31 × 10^–4^ M). These complexes were found to cause nuclear chromatin cleavage, as observed by AO/ EB staining assay and gel electrophoresis of pBR332 DNA cleavage in the presence of sodium ascorbate. They bind to calf thymus deoxyribonucleic acid (CT-DNA) by intercalation. (27) exhibited the highest interaction with CT-DNA, followed by (28) and (29). This binding to DNA led to the induction of apoptosis in BEL-7402 cells (Li et al. 2013). Apoptosis or programmed cell death can be induced by copper-based compounds containing isatin di-imine, (30) and (31). These complexes were tested on the cell lines SH-SY5Y, M14, and U937, and a mitochondria-dependent apoptosis pathway was detected. It was found that (30) with a lower penetration generated ROS and induced oxidative stress, while (31) with a higher penetration rapidly accumulated and damaged nuclear and mitochondrial components (Filomeni et al. 2007). The structure of the aforementioned copper(II) complexes is represented in Fig. 3.

In a study of copper(II) complexes formed from phen, stable bis-phen complexes were found to exhibit nuclease activity when exposed to reducing agents and molecular oxygen (Sigman et al. 1979). Here, the bis-phen complex (32) was identified as a compound capable of oxidatively degrading DNA and RNA by targeting the sugar groups (Sigman et al. 1996). Moreover, it has exhibited intriguing clinical effects, including antitumor, antifungal, antimycobacterial, and antimicrobial properties (Saha et al. 2004). In another study, Zhou et al. stated that (32) induced apoptosis specifically in the G_1_ phase of liver carcinoma cell line Bel-7402 (Zhou et al. 2002). Furthermore, Cai et al. confirmed that the apoptosis process in Bel-7402 cells upon treatment with this complex could be triggered by an excess of copper-induced through the lipophilic phen ligand (Cai et al. 2007). In the presence of intracellular reducing agents, this excess copper leads to an enhanced generation of ROS and a decrease in the amount of GSH compared to oxidized glutathione (GSSG). In addition, (32) showed a strong cytotoxic effect on human leukemic HL60 cells and human gastric cancer SGC-7901 cells (Zhang et al. 2004). However, there are limitations to the utilization of copper-phen compounds. Under physiological conditions, the formation of these complexes is harmful due to the low association constant of the second phen ligand. In addition, (32) shows low selectivity toward DNA sequences without specificity for nucleotides. To circumvent these limitations, Pitié et al. introduced a serinol bridge (clip) to connect two phen ligands and form the complexes (33) and (34) (Pitie et al. 1998a, b). These modifications offered several advantages. First, the linked phen ligands were coordinated to copper, and second, complexes (33) and (34) exhibited significantly higher DNA cleavage activity compared to (32), utilizing a pathway comparable to that of the bis-phen complex (Pitie et al. 2003; Pitie et al. 1998a, b). The increased DNA cleavage activity was attributed to structural features (Pitie et al. 2003). The incorporation of a serinol bridge was enabled the modification of copper–phenol compounds with antitumor pharmaceuticals that exhibited sequence specificity. This modification increased the selectivity of the compound toward specific DNA sequences, giving it potential anticancer properties (Zhao et al. 2023).

The development of the bifunctional complexes (35a) and (35b) was driven by an interest in improving the DNA cleavage specificity and double-strand break ability of complexes such as (34) and overcoming the drug resistance associated with cisplatin (Hoog et al. 2007). It has been suggested that protonation of the amino group in (34) facilitates binding to the polyanionic DNA structure, possibly through an interaction between hydrogen and phosphate oxygen, particularly in the minor groove (Pitie et al. 1998a, b). In vitro evaluations of the cytotoxic activities of the complexes against various human cancer cell lines revealed prominent cytotoxicity of (34), (35a), and (35b) in the L1210 cells. In particular, (35a) indicated greater efficacy than (35b) in most cases (Hoog et al. 2007; Ozalp-Yaman et al. 2008).

**Fig. 4:**
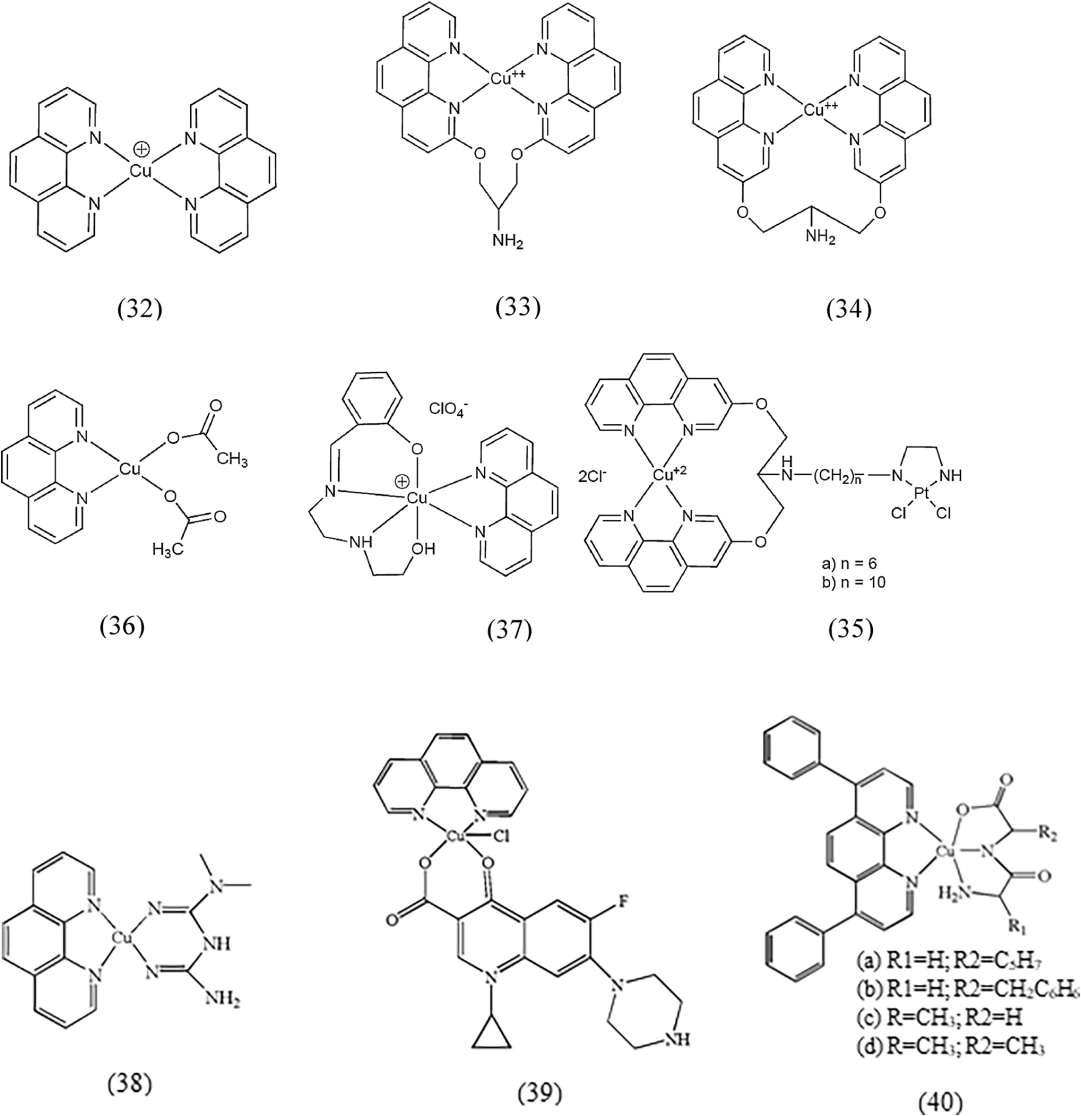
Structure of copper(II) complexes containing 1,10-phenanthroline

In a separate study, Devereux et al. investigated the structure–activity relationships (SARs) of copper complexes with two nitrogen atoms attached to the copper center. They synthesized a group of copper(II) carboxylate compounds, some of which contained chelating ligands such as phen or bipy. These complexes showed weak catalase mimetics in the presence of imidazole and were inactive without it. However, they showed excellent SOD mimetic activity. Compound (36) was demonstrated to be a potent inhibitory agent in vitro against cell lines HepG, A498, and A549, and its cytotoxicity was about sevenfold greater than that of cisplatin. Derivatives of this compound had a moderate level of solubility and exhibited inhibitory activity comparable to those of copper(II) complexes containing bis-phen. The lack of a relationship between SOD and cytotoxicity suggests that mechanisms other than mimicking SOD are involved in the cytotoxic activity of these complexes (Deegan et al. 2007a, b; Devereux et al. 2007). Rajendiran et al. synthesized and characterized a series of mixed ligand copper complexes with Htdp as the tetradentate ligand and N–N as phen, bpy, tmp, and dpq (Rajendiran et al. 2007). The complex (37) showed a six-coordinated geometry around the copper(II) center. The dpq and phen ligands partially intercalated into DNA base pairs in the minor groove, while the tmp complex interacted hydrophobically with DNA by its CH_3_ groups. The phen and dpq complexes showed higher efficiency in cleaving DNA when combined with ascorbic acid as a reducing agent. These results highlight the structural and functional properties of these copper complexes, especially their potential in DNA-related processes. In 2022, Alem et al. reported the significant inhibitory activity of two copper(II) complexes (38) and (39) containing phen against MCF-7 cells. The cytotoxicity of (38) (IC_50_ = 4.29 µM) and (39) (IC_50_ = 7.58 µM) proved to be stronger than cisplatin (IC_50_ = 18.62 µM) against the mentioned cells. Molecular docking analysis also agrees well with the experimental tests, with binding affinities of –7.35, –8.76, and –6.32 kcal/mol, respectively, for (38), (39), and cisplatin toward ERα (Alem et al. 2022). In 2023, Fernández et al. investigated the DNA binding of copper(II) complexes of dipeptide-bathophenanthroline (40) and demonstrated strong cytotoxicity for these compounds against the tumor cell lines MDA-MB-231, MCF-7 and A549 (Fernández et al. 2023). The structure of the aforementioned copper(II) complexes is shown in Fig. 4.

### Mechanisms of action

#### Apoptosis

Copper is tightly regulated in the body and is not normally found as an independent ion as it is usually bound to proteins. Proteins that require copper ions for their function are important for copper transport and cancer treatment. The copper-containing SODs are a diverse group of enzymes that play a role in the degradation of ROS by converting O_2_^•–^ into O_2_ and H_2_O_2_ through a process called disproportionation. ROS are harmful compounds that are formed during the body's oxidation reactions and contribute significantly to the progression of diseases, including cancer (Cheung and Vousden 2022). Cancer cells provide a balance between ROS and antioxidants, which affects certain signaling pathways involved in carcinogenesis. Factors, such as pressure, UV radiation, ischemia/reperfusion, and inflammation, can cause overproduction of ROS, leading to oxidative stress.

Ferredoxin-1 (FDX1) is a key component in cell death induced by copper exposure. It acts as a carrier that converts copper(II) to (I) and triggers cell death. It was identified as a regulator at an earlier stage and controls the modification of proteins with lipoic acid during cell death induced by copper and tricarboxylic acid (TCA) cyclin (Tsvetkov et al. 2022). In reduction-activated signaling, ROS combine with regulatory signals and initiate redox mechanisms that stimulate autophagy and apoptosis in cancer cells. Oxidative stress occurs when there is an imbalance between the generation and degradation of oxygen free radicals, which may indicate certain diseases, including cancer (Sies et al. 2022). While low levels of ROS are involved in signaling and regulate various cellular processes under normal conditions, high levels of ROS can lead to oxidative stress that damages lipids, proteins and DNA and triggers apoptosis (Nakamura and Takada 2021).

Excess copper in cells can generate a large amount of ROS, leading to cytotoxicity and activating multiple apoptotic pathways. Copper is involved in Fenton and Haber–Weiss reactions and catalyzes the creation of highly reactive^**.**^OH radicals and lipid peroxidation of membranes (Nakamura and Takada 2021). Exploiting copper's ability to trigger cancer cell death may offer potential strategies for the advancement of cancer treatments. Excess copper not only induces oxidative stress but also causes stress in the ER, leading to DNA damage and inhibition of cell proliferation (Oe et al. 2016). In tumor cells, various intrinsic and extrinsic cellular stress factors disrupt protein homeostasis in the ER, which can shift regulatory signals from adaptive to proapoptotic, leading to cell apoptosis. Copper increases ER permeability, inhibits ER corrective capacity, and increases ROS production, leading to proapoptotic signaling and the elimination of damaged cells (Wei and Fang 2021; Gul et al. 2020). In a previous study by our group on a pyridine-2,6-dicarboxylate copper(II) complex, apoptosis induction was confirmed after ROS generation and cell cycle arrest (in the G_2_–M phase) (Abdolmaleki et al. 2023a, b).

Regulation of the cell cycle is critical for maintaining cell proliferation and preventing tumor development. An interruption of the cell cycle can cause cell cycle arrest, which inhibits cell proliferation and induces apoptosis (Liu et al. 2022a, b). The cell cycle is a tightly regulated process, and abnormalities in cell cycle regulation can contribute to tumor cell proliferation. Gaining insights into the mechanisms of cell cycle regulation has important implications for the prevention and treatment of diseases associated with cell cycle dysregulation. Stages G_1_–S, S, and G_2_–M serve as important checkpoints in the cell cycle. Cell cycle arrest plays a critical role in the inhibitory potency of many cytotoxic compounds on tumor cell growth (Vakili-Samiani et al. 2022). NSC319726 has been introduced as a strong inhibitor of cancer cell cycle progression. Research has shown that dysregulation of copper can cause DNA damage resulting in cell cycle arrest (Shimada et al. 2018). The combination of NSC319726 with metal ions, especially copper ions, enhances its inhibitory effect on cancer cells. The binding of copper to NSC319726 triggers the production of ROS in cells and the degradation of deoxyribopurines, leading to cell cycle arrest in the G phase and apoptosis. These findings indicate that copper-mediated oxidative stress and DNA damage are major consequences of cell cycle arrest (Ji et al. 2023).

In 2021, it was shown that two tetragonal pyramidal copper(II) complexes containing benzimidazole derivatives have the potential to bind to DNA through insertion and groove binding. These compounds were able to cleave CT-DNA via an ascorbic acid-mediated pathway that relies on singlet oxygen. Both complexes showed significant inhibitory activity against A549, HeLa, and SGC-7901 cancer cells. The complex containing 5-chloro-2-(2′-pyridyl)benzimidazole showed higher cytotoxic activity compared with the complex with 2-(2′-pyridyl)benzimidazole as ligand, which correlated with its stronger binding and cleavage ability to DNA, suggesting that the inhibitory potency may be due to DNA binding. The mechanistic study performed on cells showed that the complexes arrested the cell cycle in the G_2_/M phase, enhanced intracellular ROS levels, and decreased mitochondrial membrane potential )MMP(. It was concluded that the complexes can induce apoptosis by causing DNA damage and disrupting mitochondrial function through the generation of ROS. Moreover, an in vivo study showed that the complex with 5-chloro-sunstitution suppressed the growth of tumors by 50.44%, outperforming the efficacy of cisplatin (40.94%) (Cai et al. 2021). In another study, Xu et al. concluded that a copper complex containing disulfiram (DSF), a drug known to make cancer cells dependent on copper and known to have deleterious effects on their viability, induces apoptosis and cell cycle arrest in multiple myeloma (MM) cells. They also observed disruption of mitochondrial membrane integrity and activation of apoptotic signaling pathways. In animal models, DSF/Cu demonstrated the ability to reduce tumor volume and improve overall survival. These results underline the promising potential of DSF/Cu as a suitable therapeutic compound for MM (Xu et al. 2020).

#### Angiogenesis

Angiogenesis is regulated by a complex interplay of various stimulating and inhibitory factors. The most important signaling system involved in the proliferation and migration of endothelial cells, which form the basis of blood vessels, is the vascular endothelial growth factor (VEGF) and its receptors. This system is crucial for the development of the embryonic vascular system and is also activated during neoangiogenesis in tumor growth. The biological effects of the VEGF system on cells depend on the presence of various factors and receptors in the tissue, in particular the ratio of the different isoforms of the VEGF. Other signaling systems, such as notch ligands Delta-like 4 (Dll4/Notch), play a role in the selection of endothelial cells for angiogenic expansion. Vascular stabilization and maturation involve the formation of the vessel wall, which is regulated by the PDGFB/PDGFRbeta signaling system and the angiopoietins (Ang1, Ang2) and their receptor tyrosine kinase with Tie2, which recruit mural cells, such as pericytes and smooth muscle cells (Shi et al. 2023).

Angiogenesis is a crucial process for tumor progression and metastatic spread driven by copper-dependent angiogenic factors and enzymes (Hariprabu et al. 2021). Tumors can upregulate copper-related signaling pathways to promote angiogenesis. Although tumor immunotherapy shows promise, it is only effective in certain patients and may only have a transient effect (Yang 2015). The copper deficiency can inhibit angiogenesis by blocking the expression of VEGF and impairing tumor blood vessel formation, effectively starving the tumor. In 2024, Wang et al. synthesized a series of new copper complexes, including mono-, bi-, and tri-nuclear complexes with thiophene-2-formaldehyde TSC and a tetra-nuclear complex derived from 1,2,4-triazole, to develop more effective metal agents to inhibit tumor growth. They investigated the SARs of these complexes. The tri-nuclear copper complex showed the highest inhibitory activity against T24 cells compared to the other copper complexes. In vivo studies showed that this complex had a better antitumor effect than cisplatin and fewer side effects. Further studies on the mechanism of action showed that the copper complexes induced apoptosis in cancer cells and inhibited tumor angiogenesis by preventing migration and invasion of vascular endothelial cells, arresting the cell cycle in the G_1_ phase, and inducing autophagy (Wang et al. 2024). In another study, a modified phen–copper(II) complex, CPT8, containing a triphenylphosphonium group attached to an alkyl chain, showed potent antiproliferative activity against various cancer cells, including TNBC and MDA-MB-231 cells. CPT8 induced mitophagy by activating the PINK1/Parkin and BNIP3 signaling pathways in cancer cells, which was primarily due to mitochondrial damage. Specifically, CPT8 reduced the ability of human umbilical vein endothelial cells (HUVEC) to form tubes by downregulating Nrf2. The antiangiogenic potential of CPT8 was confirmed by the decreased expression of VEGF and CD34 in HUVEC. In addition, CPT8 inhibited the formation of vasculogenic mimicry by suppressing the expression of vascular endothelial cadherin and the matrix metalloproteinases MMP2 and MMP9. CPT8 also attenuated the metastatic potential of MDA-MB-231 cells. In vivo studies showed that CPT8 suppressed tumor proliferation and vascularization, as indicated by the downregulation of Ki67 and CD34 expression. This makes CPT8 a promising metallic drug candidate for the treatment of TNBC (Zheng et al. 2023). Unver et al. reported that a copper(II) complex containing ImCF3 and bipy as ligands exhibited significantly potent antiangiogenic potential. The complex showed higher antiangiogenic activity (mean = 1.06 ± 0.01) compared to the standard antiangiogenic drug ( ±)-thalidomide (mean = 0.8 ± 0.2). In addition, it showed no irritation or embryotoxicity. However, both ligands showed weak antiangiogenic effects. In particular, one of the ligands, ImCF3, showed a significant irritation potential (86 ± 20%) at a concentration of 50 µg/pellet compared to the standard irritant SDS (84 ± 10%) on the CAM assay (Ünver et al. 2022). In another study, the antiangiogenic properties of copper(II) complexes with 1-adamantoylhydrazone with pyridine rings were investigated. The results confirmed that all three copper(II) complexes can inhibit angiogenesis in vascular endothelial cells (Rodić et al. 2016). In general, studies confirm that copper-based compounds involved in the regulation of angiogenesis could lead to the development of drugs that can inhibit or stimulate angiogenesis in various pathological conditions (Wang et al. 2024; Zheng et al. 2023; Ünver et al. 2022).

#### Cuproptosis

Cuproptosis is a recently discovered type of cell death caused by the accumulation of copper. Tsvetkov et al. made this discovery in 2022 while investigating the anticancer effects of the copper ionophore elesclomol (ES). They observed that copper interacts with a mitochondrial enzyme called FDX1, leading to an increase in ROS, which ultimately leads to cell death (Tsvetkov et al. 2022). Cuproptosis is characterized by the aggregation of lipoylated mitochondrial enzymes and a loss of iron-sulfur proteins. The excess copper is transported into the mitochondria via ionophores, and the reduction from copper(II) to (I) leads to the aggregation of lipoylated proteins and destabilization of iron-sulfur cluster proteins, which ultimately triggers cuproptosis (Wang et al. 2022; Xie et al. 2023). It is worth noting that cuproptosis differs from other known forms of cell death, such as apoptosis, necroptosis, pyroptosis, and ferroptosis. Unlike these forms, cuproptosis is not dependent on ROS and cannot be inhibited by antioxidants. In addition, cuproptosis is linked to mitochondrial function and energy metabolism. Cancer cells that rely on mitochondrial respiration are more susceptible to cuproptosis than cells that rely on glycolysis. Overall, cuproptosis sheds new light on the mechanisms behind copper-induced toxicity and cell death. The discovery of cuproptosis has prompted researchers to investigate the role of copper in cancer development and its potential as a therapeutic target. By harnessing the toxicity of copper, it may be possible to kill cancer cells. Copper-based compounds, both new and repurposed, can be used in combination with existing anticancer drugs or as a starting point for further optimization of treatment (Tsvetkov et al. 2022; Wang et al. 2022). Cuproptosis has attracted considerable interest in cancer research due to its potential to inhibit tumor cell proliferation, reverse drug resistance, and provide novel therapeutic approaches similar to ferroptosis. Promising compounds have been identified that can promote cuproptosis in preclinical models. In colorectal cancer, downregulation of the FDX1, DLAT, SDHB, and DLST genes in primary tumor tissues suggests a role of cuproptosis in cancer progression. Patients with higher expression of these genes in tumor tissue have a better prognosis (Yang et al. 2023a, b, c, d; Yang et al. 2023a, b, c, d; Yang et al. 2023a, b, c, d; Yang et al. 2023a, b, c, d). Treatment with ES-Cu significantly reduces the viability of colorectal cancer cells. The copper chelator tetrathiomolybdate (TTM) inhibits cuproptosis, indicating its role as a cuproptosis inhibitor. In addition, 2-deoxy-D-glucose, an inhibitor of glucose metabolism, sensitizes cancer cells to cuproptosis. Galactose and octyl itaconate (4-OI) also promote cuproptosis, with 4-OI inhibiting GAPDH-mediated aerobic glycolysis and silencing of FDX1 reversing the cuproptosis-promoting effects. In vivo experiments show that ES-Cu enhances the antitumor effect of 4-OI (Yang et al. 2023a, b, c, d; Yang et al. 2023a, b, c, d; Yang et al. 2023a, b, c, d; Yang et al. 2023a, b, c, d). Anisomycin, similar to ES and buthionine sulfoximine (BSO), inhibits the proliferation of ovarian cancer stem cells (OCSCs) by possibly promoting cuproptosis. Curcumin affects ferroptosis and cuproptosis in various HCC cells in a cell-specific manner, and analysis of single-cell transcriptome data suggests its potential as a cuproptosis inducer (Liu et al. 2023a, b). The cuproptosis-related gene CDKN2A is associated with the malignant behavior of head and neck squamous cell carcinoma (HNSCC), and plicamycin inhibits the progression of HNSCC, indicating its potential as a cuproptosis inducer (Fan et al. 2022). Sorafenib, a multi-tyrosine kinase inhibitor used in HCC treatment, and erastin, a ferroptosis inducer, enhance copper ionophore ES and ES-Cu-induced cuproptosis in HCC cells by promoting copper-dependent lipoylated protein aggregation, inhibiting FDX1 protein degradation, and decreasing intracellular GSH synthesis. These results suggest a link between cuproptosis and ferroptosis, and the combination of copper ionophores and ferroptosis inducers could be a promising therapeutic strategy for HCC (Wang et al. 2023a, b).

It can be concluded from the studies that copper complexes have the potential to induce cuproptosis depending on their composition and properties. However, it should be noted that the specific mechanisms and effects of copper complexes may vary depending on the specific complex and cell type. Further research is needed to fully understand the mechanisms and potential of copper complexes in inducing cuproptosis (Xie et al. 2023).

#### Paraptosis

**Fig. 5:**
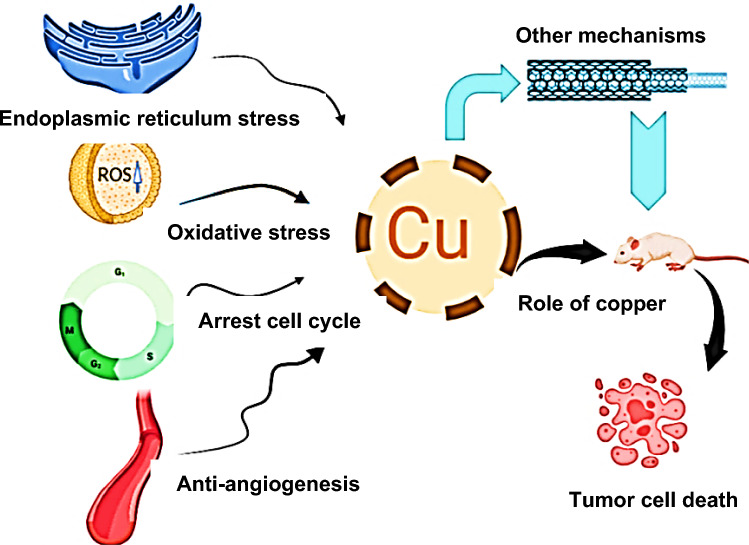
Copper-induced cell death through activation of stress pathways (ER and oxidative stress), cell cycle arrest, and anti-angiogenesis to treat cancer

Paraptosis is a form of programmed cell death that differs from apoptosis and is characterized by specific morphological changes in the cell. It was first identified by Sperandio et al. in 2000 and is known as type III programmed cell death (PCD). Paraptosis is characterized by increased cytoplasmic density, vacuolization, swelling of the mitochondria, ER, and the formation of multimembrane vesicles (Sperandio et al. 2000; Tardito and Marchiò, 2009). Finally, macrophages phagocytize the cells without causing inflammation in the surrounding tissues. Various assays, including DNA labeling and enzymatic analysis, are commonly used to study paraptosis (Ji et al. 2023). Recent research has shown that certain copper complexes can induce paraptosis in tumor cells by suppressing the activity of proteasomes and promoting the accumulation of misfolded proteins, thereby triggering ER homeostasis. This effect is comparable to the well-known chemotherapeutic agent cisplatin (Ji et al. 2023; Gandin et al. 2012). In a study, it was observed that hinokitiol–copper complex (HK–Cu) caused a significant accumulation of ubiquitinated proteins in A549 and K562 cells. Moreover, HK–Cu showed strong inhibition of 19S proteasomal deubiquitinases (DUBs) compared to its effect on the chymotrypsin-like activity of the 20S proteasome. HK–Cu effectively induced caspase-independent and paraptosis-like cell death in A549 and K562 cells. Finally, the researchers discovered that HK–Cu-induced cell death was dependent on ATF4-associated ER stress, but was not related to the generation of ROS. Overall, these results suggest that HK–Cu has the potential to inhibit the activity of 19S proteasomal DUBs and induce paraptosis-like cell death, making it a promising candidate for cancer treatment (Chen et al. 2017). Paraptosis can be triggered by different mechanisms (Hanson et al. 2023). Translation and transcription are essential for triggering this process. The ER plays an important role in paraptosis by accumulating misfolded proteins, leading to ER stress and the UPR. Inhibition of the proteasome can lead to the assembly of polyubiquitinated proteins and ER stress. The ER is connected to the mitochondria by MAMs, which help to maintain calcium balance. ER stress triggers the UPR and activates proteins like BiP and CHOP. Paraptosis occurs when the apoptosis machinery is not functioning, and one of its distinct features is the dilation of the ER (Zhao et al. 2023). In cells, the ER serves as a storage site for calcium, and IP3 receptors (IP3R) are located in the mitochondria-associated ER membranes. Disturbances in calcium levels can lead to cell death. Calcium leaves the ER via IP3R and enters the mitochondria via the MCU. The ER is responsible for protein synthesis, folding and processing, and calcium plays a role in supporting chaperone function. When calcium is deficient, chaperones are impaired, leading to an accumulation of misfolded proteins and swelling of the ER. Mitochondrial calcium overload leads to oxidative stress. There may be a control loop between ROS and calcium, whereby calcium is upstream of ROS formation and ROS influences the release of calcium. ROS also increases ER stress and thus damages both the ER and the mitochondria. Mitochondrial calcium overload disrupts the distribution of ions, reduces the potential at the membrane and leads to organelle collapse. ROS stimulates the release of carbon monoxide, activates ion channels and affects potassium and sodium levels, leading to ATP depletion and cell death. Paraptosis involves the impairment of both the ER and the mitochondria. The increased presence of mitochondrial proteins, such as ATP synthase and prohibitin, indicates the involvement of the mitochondria. Prohibitin acts as a mediator and agonist in the paraptotic death pathway and influences various cellular processes (Yokoi et al. 2022; Shubin et al. 2016). The types of copper-induced cell death mechanisms are shown in Fig. 5

### Clinical studies and translational potential

[64Cu]Cu-ATSM, is a copper complex currently being evaluated in clinical trials for its ability to represent oxygen deprivation in advanced-stage tumors in a localized area of rectal cancer (NCT03951337) and bulky tumors (NCT04875871) (Savi et al. 2017). Liu et al. reported the automated cyclotron generation and preparation of [64Cu]Cu-ATSM using a synthesis module that is readily available for commercial use (Liu et al. 2022a, b). However, it is important to note that multiple administrations of [^64^Cu]Cu-ATSM can lead to liver toxicity (Yoshii et al. 2018). To mitigate this risk, the administration of penicillamine, a heavy metal chelator, has been suggested to minimize the amount of radiation absorbed by vital organs, such as liver and small intestine (Yoshii et al. 2014).

Researchers have successfully developed and confirmed the efficacy of [^64^Cu]Cu-ATSM for diagnostic and theranostic applications (Matsumoto et al. 2019). The potential risk of chemical contamination from the degradation of [^64^Cu]Cu-ATSM is considered insignificant, even at therapeutic doses. Positron emission tomography (PET) with [^64^Cu]Cu-ATSM has been shown to provide relevant and useful information about tumor oxygenation in a clinical context to predict response to therapy and survival of patients with solid tumors (Liu et al. 2020; Xie and Wei 2022). [^64^Cu]Cu-ATSM as a theranostic agent has been exploited in Japan. Further exploration of its mechanisms of action may contribute to the understanding of its therapeutic effects (Xie and Wei 2022). The use of ^64^Cu-labeled agents offers advantages for multicenter clinical trials compared with ^60^Cu- or ^62^Cu-labeled agents because they have a longer half-life, which facilitates shipment to multiple centers. The β-decay and Auger electron emission of ^64^Cu also allows potential therapeutic applications with [^64^Cu]Cu-ATSM under appropriate conditions (McMillan et al. 2015). In tumor masses, [^64^Cu]Cu-ATSM tends to accumulate mainly in the outer periphery, i.e., in regions of the tumor where there is a lack of oxygen but active cancer cells are still present (Obata et al. 2003). [^64^Cu]Cu-ATSM has been granted Investigational New Drug status by the US Food and Drug Administration. The comparison of [^60^Cu]Cu-ATSM and [^64^Cu]Cu-ATSM in patients with cervical cancer confirmed a prominent correlation in tracer uptake, with [^64^Cu]Cu-ATSM PET images showing a better tumor to-background ratio than [^60^Cu]Cu-ATSM [142]. [^64^Cu]Cu-ATSM is better absorbed in hypoxic tissues and offers several significant advantages over other radiotracers. It may have better penetration through the blood–brain barrier (BBB) than [^18^F]FAZA tracer, which is excreted through the urinary tract (Savi et al. 2017). In addition, [^64^Cu]Cu-ATSM shows more uptake in hypoxic tissues and faster washout in normoxic cells compared to [^18^F]FMISO, which requires at least a two-hour equilibration time before scanning (Xie and Wei 2022; Takasawa et al. 2008).

**Fig. 6:**
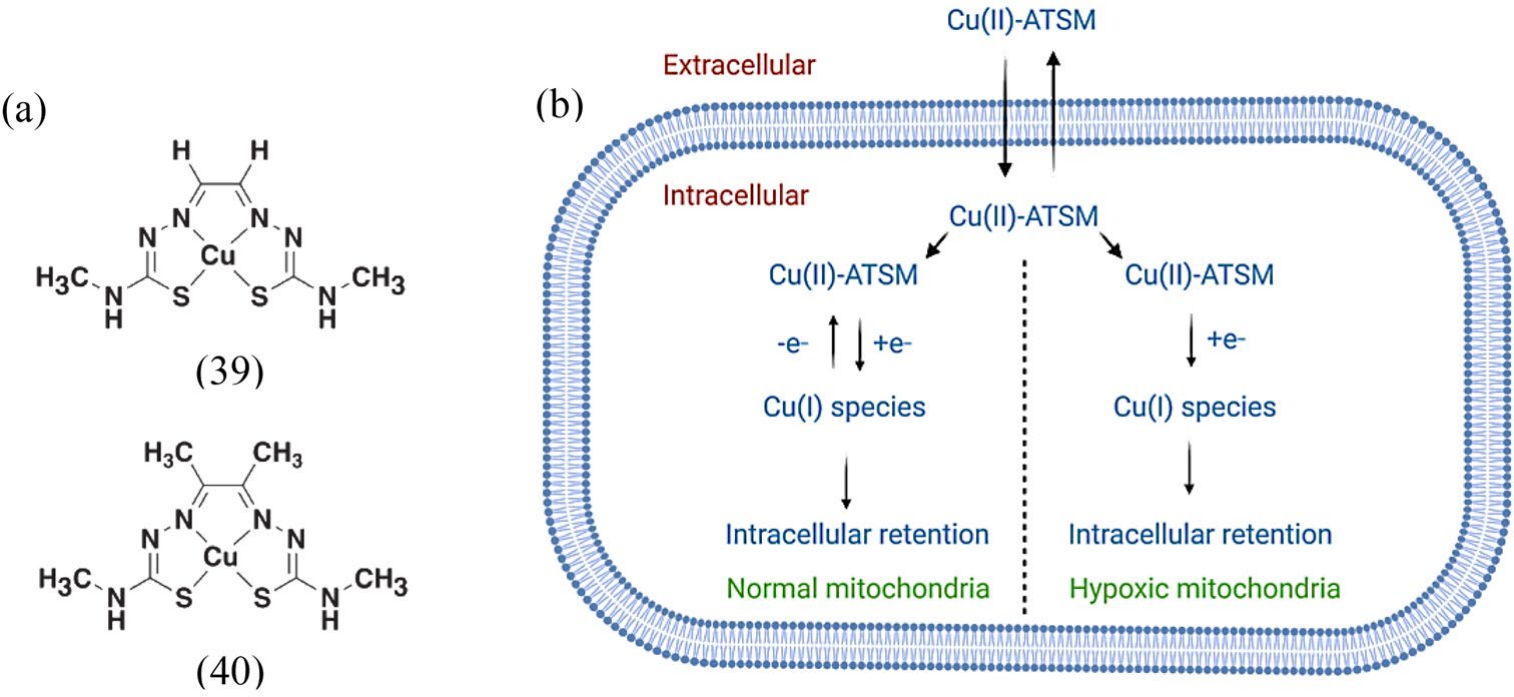
**a** Chemical structures of some copper(II) complexes used in cancer imaging and **b** mechanisms involved in cellular uptake and storage of Cu-ATSM under both normal and hypoxic conditions

The Cu(II)-GTSM (39) (Xie and Peng 2017), can transport exogenously bound copper directly into cells, activating mechanisms in which copper is a critical cofactor. On the other hand, Cu(II)-ATSM (40) is a biosimilar that releases exogenously bound copper only under conditions of low oxygen levels (Fig. 6a) (Xie and Wei 2022). Both Cu-ATSM and ATSM are rapidly eliminated from the circulation with half-lives (T1/2) of approximately 21.5 and 22.4 min, respectively (Xie and Wei 2022; Nam et al. 2022). The BBB is a highly selective gateway that regulates the movement of molecules between the systemic circulation and the brain and in the reverse direction. Copper(II)-ATSM exhibits an increased ability to penetrate and cross the BBB (Nam et al. 2022). The penetration of this compound in cells is influenced by the properties and nature of copper. The exact mechanisms of Cu-ATSM uptake have not been fully elucidated, but several possibilities have been proposed. A mechanism states that Cu(II)-ATSM can enter mitochondria and be reduced from copper(II) to copper(I), resulting in long-term storage of the metal or radiometal in hypoxic cells. Cu(II)-ATSM can easily penetrate cells due to its small size, excellent ability to penetrate membranes, and low redox potential. In cells that are too reduced, such as under hypoxic conditions, the copper(II) in Cu(II)-ATSM is reduced to copper(I). This reduction results in copper(I) being released from the ATSM and subsequently trapped in the cells (Fig. 6b) (Nam et al. 2022).

Recent research has also shown that Cu-ATSM uptake is not only indicative of hypoxia but also reflects intracellular states characterized by mitochondrial damage and over-reduction. In normoxic cells, Cu(I)-ATSM is rapidly reoxidized to Cu(II)-ATSM by molecular oxygen and subsequently eliminated from the cells (Maurer et al. 2002). Another possible pathway is the dissociation of copper(II) from copper(II) complexes and the reduction of it to copper(I) by reductases present in the blood circulation or the tumor surroundings. Tumor cells suffering from oxygen deficiency often have an increased concentration of CTR-1, which facilitates the transport of copper(I) into the cells (White et al. 2009).

Cu-ATSM labeled with copper isotopes, such as ^60^Cu, ^62^Cu, and ^64^Cu, has been used to visualize tumor hypoxia and blood perfusion. It shows significant absorption in the brain and heart with enhanced permeation under hypoxic conditions. Translational studies have demonstrated its safety profile, rapid clearance from the bloodstream, and accumulation in tumors within minutes. The penetration of Cu-ATSM depends on the oxygen pressure in the tissue, making it a valuable imaging tool for hypoxia. In different cancer types, the uptake patterns of Cu-ATSM differ from those of ^18^F-FDG, indicating different characteristics in squamous cell carcinoma (SCC) and adenocarcinoma. Cu-ATSM was also recognized to visualize regional oxidative stress in patients with MELAS, a mitochondrial disorder. Cu-ATSM has been correlated with ^15^O tracers to assess cerebral blood flow and oxygen extraction fraction in patients with cerebrovascular disease (Xie et al. 2022). Moreover, Cu-ATSM uptake is noticeably greater in grade IV gliomas compared with grade III gliomas and correlates with HIF-1α expression, a marker of hypoxia (Tateishi et al. 2013). In addition, the tumor-to-muscle activity ratio of Cu-ATSM has shown promise for predicting treatment outcomes in non-small cell lung cancer (NSCLC) and cervical cancer. To tumor hypoxia imaging, some clinical trials are investigating the safety data and initial efficacy of Cu(II)ATSM in patients with Parkinson's disease (PD) or amyotrophic lateral sclerosis/motor neuron disease (Savi et al. 2017; Xie and Wei 2022).

**Fig. 7:**
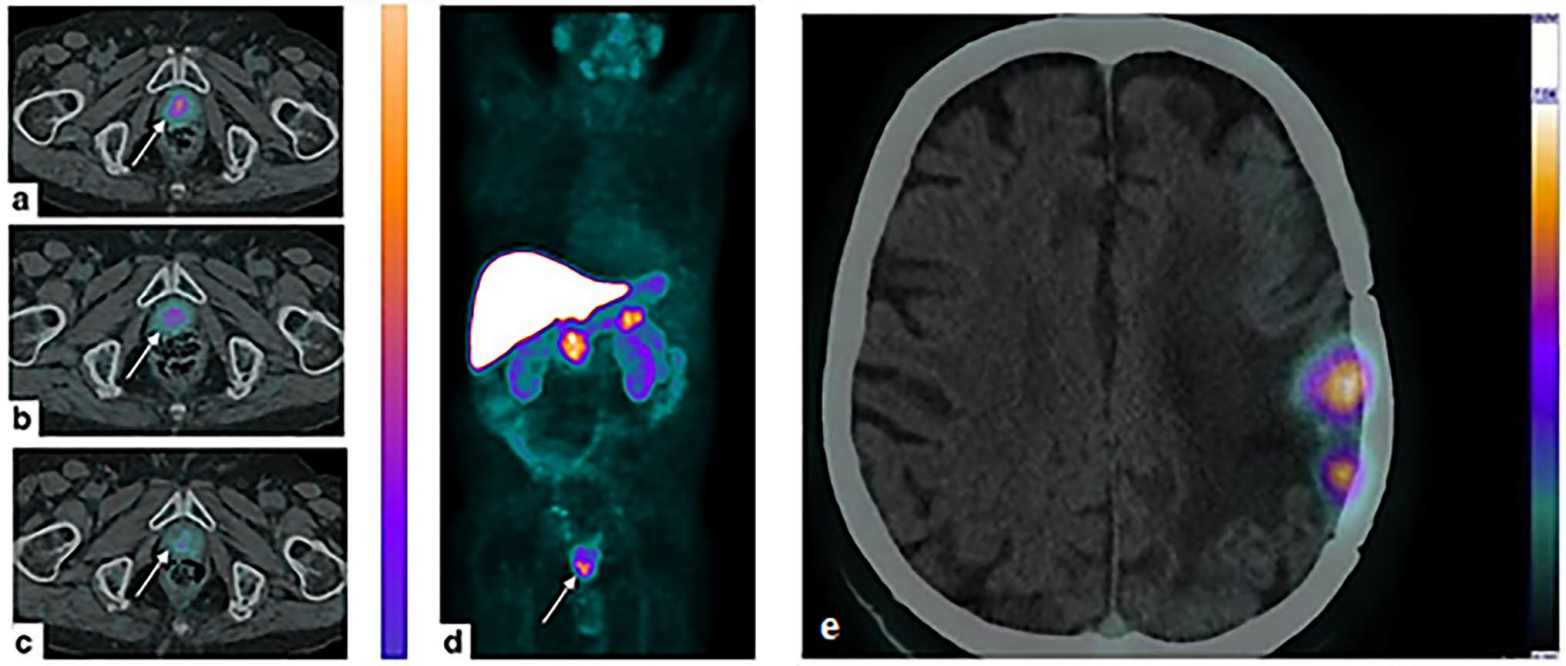
^64^CuCl_2_ PET/CT imaging: **a** Uptake of the tracer in the prostate apex, near the midline, 1 h after injection, **b** Marked reduction of tracer uptake 4 h after injection, **c** 24 h after injection, **d** Maximum intensity of tracer uptake and **e** Presence of cerebral glioblastoma in the brain, imaged 1 h after injection

Capasso et al. investigated the role of ^64^Cu-PET in assessing the stage of individuals with prostate cancer (PCa). The study included seven PCa patients, three of whom were undergoing hormone therapy. The lack of urinary ^64^CuCl_2_ excretion helped identify pelvic prostate cancer lesions. The penetration of ^64^Cu-PET showed higher values in the original tumors of patients without hormone therapy than in treated patients. Uptake in nodes varied, with a focal dose in normal-sized nodes and no noticeable absorption in suspicious lymph nodes. These initial findings suggest that ^64^Cu-PET has the potential for the diagnosis of PCa (Capasso et al. 2015). A subsequent study by Piccardo et al. examined the distribution, radiation dose, and behavior of lesions in a group of 50 patients with PCa who experienced biochemical recurrence after surgery or radiotherapy. The accuracy of the diagnosis of ^64^Cu-PET/CT, ^18^F-choline PET/CT, and multiparametric MRI (mpMRI) was compared. The research confirmed the efficacy of ^64^Cu-PET/CT in the detection of local recurrence as well as bone and lymph node metastases (Piccardo et al. 2018). The better performance of ^64^Cu-PET/CT can be attributed to its better biodistribution compared to ^18^F-choline PET/CT. Unlike choline, copper ions are not excreted by the kidneys and do not accumulate in the urinary system, allowing a more accurate assessment of the pelvic region and prostate bed with early visualization of lesions in the pelvic area. Dosimetry studies have also shown that the amount of radiation absorbed by recurrent PCa and its spread to other areas is minimal, without considering the therapeutic outcome. Righi et al. have shown that the therapeutic effect of ^64^Cu in PCa may be due to the emission of Auger electrons with high linear energy transfer rather than beta radiation. Although its efficacy may be limited in large PCa lesions, it may be effective against residual disease or micrometastases because of the damaging effects of Auger electrons released by ^64^Cu and absorbed into the cells (Fig. 7a–d) (Righi et al. 2018). In addition, ^64^Cu-PET/MRI was shown to have a higher overall detection rate than 18F-choline PET/MRI, 64Cu-PET/CT, 18F-choline PET/CT, and mpMRI alone when assessing the presence of PCa recurrence in the same area (Paparo et al. 2020). Although there are promising results regarding the use of ^64^CuCl_2_-PET/CT for the evaluation of PCa, Cantiello et al. pointed out that the technique has its limitations and is not always superior to imaging modalities currently used in PCa (Cantiello et al. 2021). There are no significant differences between mpMRI and 64CuCl2-PET/CT in the detection of metastatic lymph nodes in the initial diagnosis of primary PCa. However, in re-staging, a significantly higher detection rate is observed in lesion-based analysis than in 18F-choline PET/CT, both in local and lymph node-based staging, but not in patient-based analysis. In addition, ^64^CuCl_2_ is mainly degraded by the liver, which may fail to detect liver metastases. Mascia et al. reported a prospective study to assay the safety and efficacy of ^64^CuCl_2_ as a PET radiopharmaceutical for imaging other urologic malignancies (Mascia et al. 2021). The research included a total of 23 patients with renal, bladder, and penile cancers. Uptake of ^64^Cu was highest in PCa lesions (SUVmax 11.5), relatively high in bladder cancer (SUVmax 6.2), and lower in penile cancer (SUVmax 3.9) and renal cancer (SUVmax 5.0). This suggests that ^64^Cu-PET/CT may be beneficial to assess primary and locally recurrent bladder cancer lesions because of a significant contrast between the desired area and the surrounding background. However, the potent background uptake of ^64^Cu in the kidneys (SUVmax 10.4) may reduce its utilization in the assessment of primary renal cancer tumors (Mascia et al. 2021). In another study, Panichelli et al. investigated the feasibility of ^64^CuCl_2_ PET/CT for imaging in patients with glioblastoma (GBM). In this study, all patients with GBM had high tumor uptake of ^64^Cu, whereas patients with low-grade astrocytoma had low tumor uptake of ^64^Cu. Importantly, neoplastic tissue was rapidly and unequivocally detected and radioactivity remained stable over time (Fig. 7e). This research explained further reasons for the use of ^64^CuCl_2_ as a radiopharmaceutical for PET imaging (Panichelli et al. 2016). Fiz et al. performed a study of the use of ^64^Cu-PET/CT in pediatric patients with diffuse high-grade glioma and found desirable dosimetry and the ability to detect tumor recurrence in cases with unclear MRI results (Fiz et al. 2022). Another study by García-Pérez et al. investigated ^64^Cu uptake in individuals diagnosed with non-small cell lung carcinoma, finding high uptake in peripheral primary lung cancer lesions and nodal metastases. Patients with high ^64^Cu uptake had a partial response to chemotherapy, whereas patients with low uptake had disease progression. Expression of the CTR1 is associated with ^64^Cu uptake, indicating the potential utility of ^64^Cu PET/CT in identifying individuals who may benefit from platinum-based treatment (García-Pérez et al. 2020).

^64^CuCl_2_ is a potential radiopharmaceutical for the diagnosis and therapy of various cancers, such as PCa and GBM. PCa cells have been found to have increased uptake of ^64^CuCl_2_, leading to DNA damage and genomic instability (Guerreiro et al. 2018). In preclinical models, ^64^CuCl_2_ has shown therapeutic effects by significantly reducing the growth and viability of PCa cells. In GBM, ^64^CuCl_2_ has shown an increased affinity for tumor cells and potential as a diagnostic PET probe. In addition, it has shown therapeutic potential for GBM, with synergistic effects observed when used in combination with other treatments. Regarding copper toxicity, calculations show that the amount of copper administered during PET imaging or therapy with ^64^CuCl_2_ is not harmful to patients. The copper concentration required for cytotoxic effects is much higher than the amount administered in PET studies or therapy. The dose of ^64^Cu administered in PET imaging is below the cytotoxic threshold, and even in therapy, the dose remains below the toxicity threshold (Capriotti et al. 2022). In addition, ^64^Cu shows the possibility of radionuclide therapy in other copper-metabolizing tumors, including HCC, GBM, and MM. The results demonstrate the promising capabilities of ^64^CuCl_2_ as a valuable tool for both diagnosis and therapy in various types of cancer (Righi et al. 2018).

## Copper nanoparticles in cancer therapy

Metal-based nanoparticles have proven to be useful in various therapeutic areas due to their unique properties and synergistic effects. Copper nanoparticles have gained special attention in this regard as ideal candidates for cancer therapy. These nanoparticles have remarkable properties, including a large surface area to volume ratio, excellent compatibility with living organisms, and the ability to generate ROS when exposed to an acidic tumor microenvironment (Kang et al. 2023; Amatya et al. 2023; Liu et al. 2021a, b, c).

One of the main advantages of copper nanoparticles in cancer therapy is their capacity to specifically target cancer cells while preserving healthy cells. The targeting approach can be achieved by functionalizing the surface of the nanoparticles with specific ligands or antibodies that can recognize and bind to cancer cells. The targeting mechanism allows therapeutic agents to be delivered directly to the tumor, minimizing off-target effects (Liu et al. 2023a, b; Shen et al. 2022). Several studies have shown that copper nanoparticles can be used as effective agents in chemodynamic therapy (CDT) (Liu et al. 2021a, b, c; Liu et al. 2023a, b; Shen et al. 2022). This is a relatively new method in cancer therapy that utilizes ROS generated by the catalytic action of transition metal-based nanomaterials. Unlike traditional chemotherapy, which relies on cytotoxic drugs, CDT uses the intrinsic properties of the nanomaterials to induce tumor cell death. CDT is a promising treatment strategy for cancer that utilizes the in situ Fenton reaction, which is activated by endogenous substances, such as GSH and H_2_O_2_ without the need for external energy input (Yu et al. 2021). CDT overcomes the limitations of light penetration into tissues and has gained attention in recent years. Copper-based substrates have been developed that generate H_2_O_2_ internally and function effectively in weakly acidic tumor microenvironments (TME) (Liu et al. 2021a, b, c). In a study, copper peroxide nanodots were used to improve CDT. These nanoparticles self-supply H_2_O_2_ and release copper ions in an acidic tumor environment. The released copper ions react with H_2_O_2_ to produce^**.**^OH, which can induce cell death. The nanoparticles also accumulate in tumors and successfully inhibit tumor proliferation with minimal side effects. This study introduces a new method to prepare metal peroxide nanomaterials and offers a promising strategy to improve CDT efficacy (Lin et al. 2019).

**Fig. 8:**
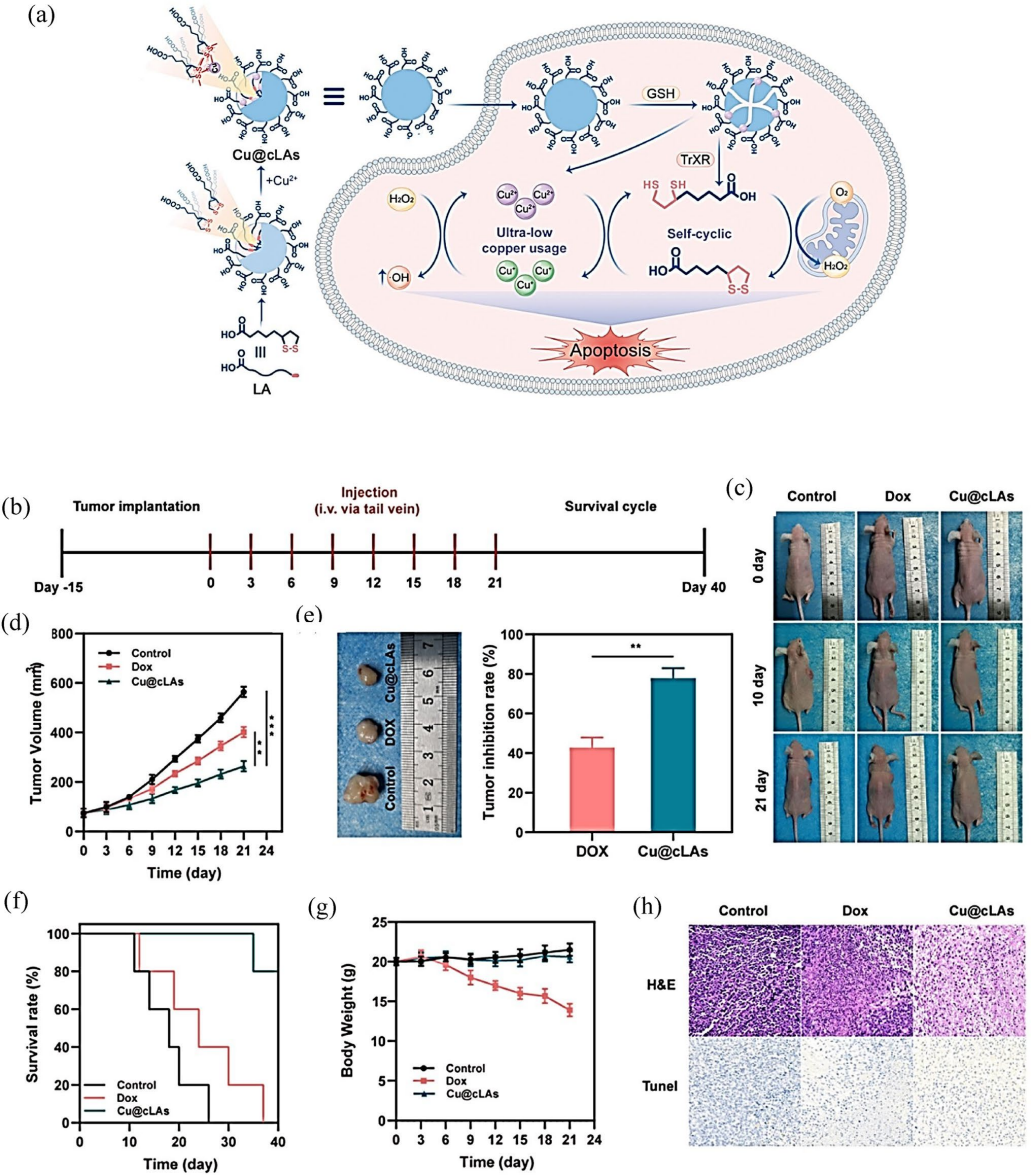
**a** Graphical representation of the self-cycling system based on Cu@cLAs for copper-mediated chemotherapeutic drug delivery. **b** Schematic of the process of MCF-7/R tumor xenograft preparation, drug delivery, and survival tracking. **c** Images showing the different sizes of tumors in mice subjected to different treatments. **d** Graphical representation of the change in tumor volume during the treatment period. **e** Digital photographs of the tumors of mice bearing MCF-7/R tumors after 21 days of therapy, along with the percentage of tumor inhibition for each treatment group. **f** Percentage of survival of mice after different treatments. **g** Changes in body weight of the mice during therapy and **h** Histological analysis of tumor sections from mice with MCF-7/R tumors, involving the use of H&E staining and TUNEL staining

In 2022, copper–iron peroxide nanoparticles (CFp NPs) were developed as a new CDT system for synergistic therapy in TME. These CFp NPs release H_2_O_2_, copper ions, and iron ions under the slightly acidic conditions of the TME. Copper and iron ions, especially at lower oxidation states, work together to efficiently generate^**.**^OH. This unique synergism, aided by the copper(I)-assisted conversion of Fe^3+^ to Fe^2+^, distinguishes this CDT system from previous systems. Remarkably, this approach achieves almost complete tumor ablation with a minimal treatment dose, without the need for additional therapies. In addition, CFp NPs can generate O_2_ during the catalysis process and show TME-dependent T1 contrast enhancement in magnetic resonance imaging. These properties are beneficial for alleviating hypoxia or monitoring tumors in vivo (Koo et al. 2022). In 2023, researchers developed another copper-based CDT agent called nanoparticle Cu@cLAs, which effectively addresses the issues of high copper consumption and potential toxicity associated with previous copper-based CDT agents. Cu@cLAs consist of copper anchored on cross-linked lipoic acid nanoparticles. When Cu@cLAs are taken up by tumor cells, they release copper (I) and (II) ions upon dissociation into lipoic acid and dihydrolipoic acid. These copper ions undergo a self-recycling process within the cells that leads to efficiently killing cancer cells by delaying the loss of copper metabolism and enhancing ROS levels (Fig. 8a). The efficacy of this self-recycling process was confirmed by sustained high copper/dihydrolipoic acid (DHLA) content and sustained ROS generation. To investigate the therapeutic efficacy of Cu@cLAs, an animal model of MCF-7/R in nude mice was used. Cu@cLAs were injected intravenously at a dose of 5 mg/kg, which corresponds to 10% of the safe dose (Fig. 8b). During the 21-day treatment process, the control groups treated with saline and the chemotherapeutic agent doxorubicin (DOX) showed rapid tumor growth, while the tumor size increased only slightly in the Cu@cLAs group (Fig. 8c and d). Further validation of the superiority of Cu@cLAs in tumor therapy was provided by the examination of removed tumors (Fig. 8e). The results showed that Cu@cLAs had higher efficacy in tumor suppression compared to DOX. Importantly, the copper content of Cu@cLAs at 5 mg/kg was significantly lower than the previously reported optimal counterparts. The therapeutic efficacy of Cu@cLAs was also confirmed by histological staining of tumor sections, which showed greater apoptosis and necrosis compared to DOX-treated mice (Fig. 8h). Consistent with the therapy results, the survival experiment showed a higher survival rate in mice injected with Cu@cLAs compared to saline- and DOX-treated mice (Fig. 8f). The Cu@cLAs-treated group exhibited a favorable level of biosafety throughout the treatment, as evidenced by the absence of significant weight changes (Fig. 4g). In general, Cu@cLAs showed an antitumor effect of up to 77.9% at a copper dose lower (0.05 mg/kg) than the normal serum copper level (0.83 ± 0.21 mg/kg). This research not only represents a promising clinical strategy to reduce excessive copper consumption in copper-mediated CDT but also provides insights for other metal-mediated therapies with high metal consumption (Cui et al. 2023).

Copper nanoparticles can also be used in phototherapy. Phototherapy includes two main approaches: photothermal therapy (PTT) and photodynamic therapy (PDT). PDT is a noninvasive treatment method that uses photosensitizers (PS) to generate cytotoxic ROS in premalignant and neoplastic conditions. In contrast, PTT focuses on inducing cell death by hyperthermia using photoabsorbing agents (Zhuo et al. 2023). PDT uses light, O_2_, and PS to produce cytotoxic ROS that trigger apoptosis in cancer cells by type I and/or type II photochemical reactions (Zhuo et al. 2023; Cheng et al. 2023). In type I reactions, the excited triplet state of PS interacts directly with cancer cell biomolecules, resulting in the formation of radical cations or anions, which then react with O_2_ to generate cytotoxic ROS (O^2−^, ^−^OH, H_2_O_2_, etc.). In type II reactions, the excited triplet state of PS sensitizes O_2_ and produces singlet oxygen (^1^O_2_) within cancer cells (Cheng et al. 2023). Although PDT shows promise in the treatment of superficial tumors, there are challenges in achieving high tumor selectivity, efficient ROS production, and monitoring treatment success. Copper-based nanomaterials offer potential solutions to these limitations. For example, copper-doped carbon dots (Cu-CDs) developed by Wang et al. exhibit high fluorescence quantum yield, low cytotoxicity, and efficient ^1^O_2_ production, effectively inhibiting the growth of multicellular spheroids (Wang et al. 2019a, b). Guo et al. designed tumor-selective nanoparticles (Cu(ii)Chl-HA NPs) that replace magnesium(II) in chlorophyll to enhance ^1^O_2_ production and achieve receptor-mediated targeting in CD44-overexpressing cancer cells (Guo et al. 2019).

**Fig. 9:**
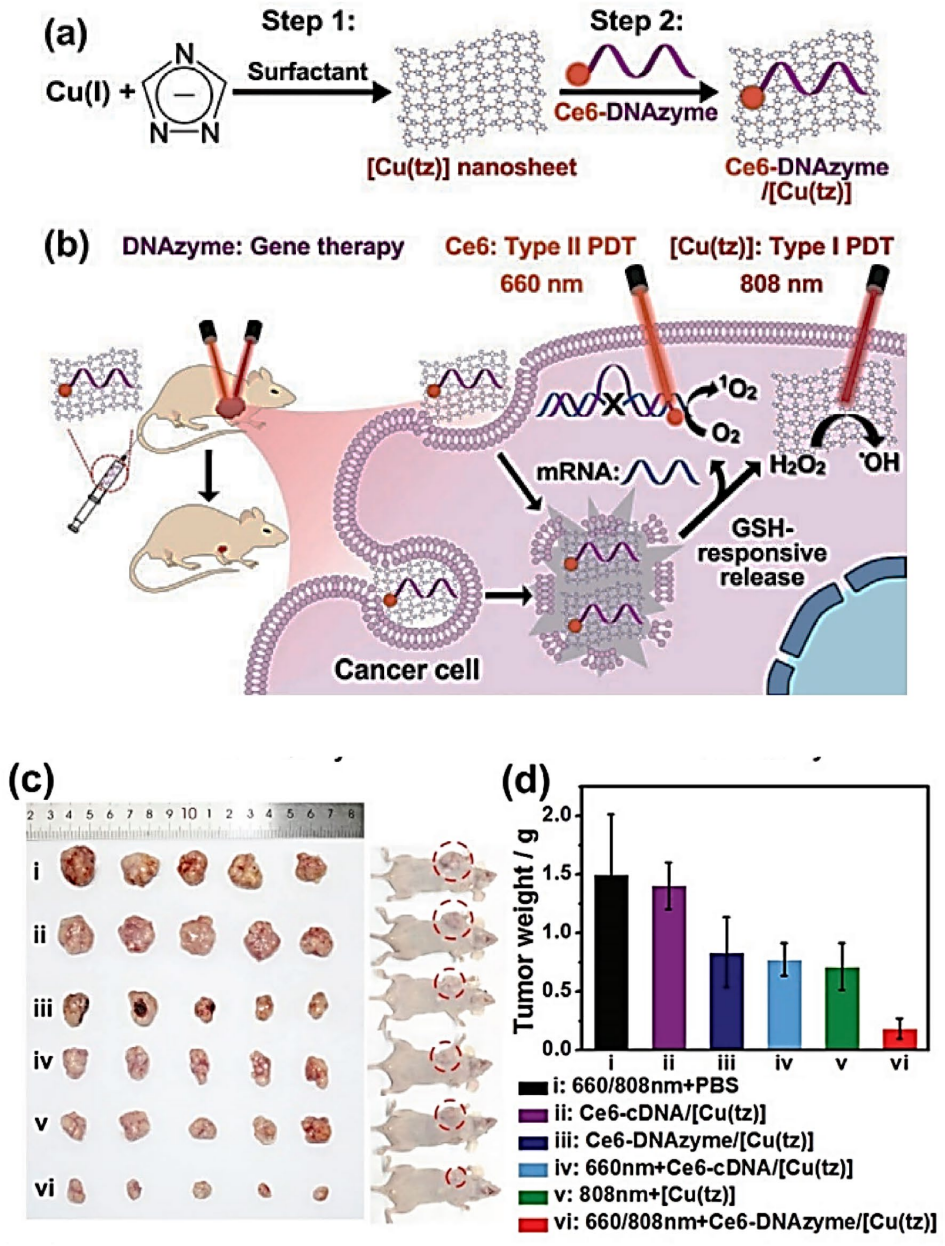
**a** Process for the preparation of 2D [Cu(tz)] nanosheets and the Ce6 DNAzyme/[Cu(tz)] therapeutic platform. **b** Schematic representation of the proposed combination therapy with DNAzyme-based gene silencing, Ce6-based PDT type II and [Cu(tz)] nanosheet-based PDT type I. **c** Photographs and **d** weights taken and recorded for analysis

Researchers have developed copper-based complexes that combine type I and type II PDT with gene silencing to overcome limitations in ^1^O_2_ yield and selectivity for cancer. For example, Liu et al. developed an ultrathin 2D copper(I)-1,2,4-triazolate coordination polymer nanosheet (Ce6-DNAzyme/[Cu(tz)]) that responds to GSH and light. This nanosheet carrier released a tumor-targeted DNAzyme that generated ^1^O_2_ through type II PDT and triggered a type I response for effective antitumor activity in hypoxic TMS (Fig. 9a and b). To evaluate the efficacy of the treatment, the nanosheet was administered intravenously to mice with MCF-7 tumors. The body weight of the mice and the relative tumor volume were determined every three days. A gradual increase in body weight of all mice was observed as time progressed, indicating minimal side effects of Ce6-DNAzyme/[Cu(tz)] in vivo. The relative tumor volumes of mice in the *phosphate-buffered saline* (PBS)-treated group with 660 nm and 808 nm laser irradiation (group i) and the Ce6-cDNA/[Cu(tz)] group in the dark (group ii) increased around 14-fold over the 21-day treatment, indicating negligible effects of laser irradiation alone or Ce6-cDNA/[Cu(tz)] injection. In contrast, gene silencing was the only method used (group iii), type II PDT alone (group iv) and type I PDT alone (group v) only partially inhibited tumor proliferation. However, in group vi, in which the combination of gene silencing, type II PDT, and type I PDT was administered, tumors regressed markedly. These findings are in agreement with the images and weights of the removed tumors from the mice that were euthanized after 21 days of treatment (Fig. 9c, d). In comparison to the tumor weights in group i, at day 21, tumors in groups iii, iv, and v treated with single therapies had decreased by 44.7%, 48.7%, and 52.7%, respectively. In contrast, the combination therapy in group vi inhibited tumor proliferation by 88.0%, demonstrating the enhanced efficacy of the combination of gene therapy, type II PDT and type I PDT (Liu et al. 2021a, b, c). Xu et al. developed glucose oxidase (GOx)-loaded copper nanoparticles (GOx@[Cu(tz)]), which triggered cuproptosis-mediated synergistic photodynamic therapy in bladder cancer. GSH stimulation initiated GOx activity, which degraded glucose and GSH and sensitized tumor cells to GOx@[Cu(tz)]-associated cuproptosis through elevated intracellular H_2_O_2_ (Xu et al. 2022). Chen et al. introduced copper-cysteamine (Cu-Cy) nanoparticles as a novel generation of PDT sensitizers activated by X-rays. These nanoparticles inhibited the proliferation and migration of cancer cells without apparent toxicities in vivo, as demonstrated by the downregulation of proliferating cell nuclear antigen (PCNA) and E-cadherin expression (Chen et al. 2022). In addition, He et al. evaluated a PDT treatment with copper-doped calcium phosphate nanoparticles (CCPCA NPs) that reduced cancer hypoxia and continuously delivered oxygen generated by catalase. This approach improved the concentration of protoporphyrin IX (PpIX) and metabolic half-life by reducing PpIX efflux and increasing PpIX formation (He et al. 2022).

Photothermal therapy (PTT) is an effective treatment method for inhibiting tumor growth using a near-infrared (NIR) laser to generate hyperthermia and selectively destroy cancer cells. Copper sulfide nanoparticles (CuS NPs) have been developed for nucleus-targeted PTT, which allows direct targeting of cancer cells and induces apoptosis through NIR irradiation (Li et al. 2018). In another study, a NIR-activated CuS nano-platform (CuS-RNP/DOX@PEI) was presented to deliver Cas9 RNP and DOX for synergistic antitumor therapy and enhance the PTT effect by accelerating the release of therapeutic agents (Chen et al. 2021). Copper-based PTT has also been shown to enhance radiotherapy (RT). A CuS-based nano-platform significantly delayed tumor growth and improved survival when combined with RT (Aishajiang et al. 2023). Furthermore, PTT can be combined with immunotherapy, as shown by a strategy using CuS-RNP@PEI, which triggers PTT and promotes factors of the immune response (Yan et al. 2021).

Copper nanoparticles have also shown success in destroying cancer tissue by hyperthermia. This method is a local anticancer treatment in which cells are exposed to high temperatures. It has shown promising results when used in combination with radiotherapy or chemotherapy for various tumor types. Researchers are continually working to improve hyperthermia by studying its effects on DNA repair pathways, immune response, and its ability to target specific tumor areas. Recent studies have found that hyperthermia can inhibit DNA repair pathways and increase tumor cell killing when combined with ionizing radiation. It has also been demonstrated that hyperthermia at 42 °C can induce an immune response and inhibit tumor growth and metastasis. Comparisons of heating methods have shown that microwave heating is more effective at killing cancer cells and increasing the release of HSP70, a heat shock protein. These results contribute to the advancement of knowledge about the effect of hyperthermia on tumor cells and provide valuable insights for further research and clinical application (Crezee et al. 2021).

Hyperthermia is a promising clinical treatment that, when used in combination with other therapies, has shown significant benefits in improving tumor control and disease-free survival without increasing side effects. Researchers have focused on determining the dose–response relationship of hyperthermia and have found that tumors with a pathologic response to preoperative radio(chemo)therapy and locoregional hyperthermia have higher tumor temperatures (Datta et al. 2015; Unsoeld et al. 2020). In 2022, Gajare et al. presented two nanomagnetic copper complexes, [Nano-magnetite-Lys@Cu(PPh_3_)I] and [Nano-magnetite-Arg@Cu(PPh_3_)I], with sizes of approximately 12 nm and 15 nm, respectively. These compounds showed notable anticancer effects toward MCF-7 cells with IC_50_ values of 104.38 μg/mL and 95.81 μg/mL, respectively, compared with the reference drug cisplatin (IC_50_ = 88.91 μg/mL). Hyperthermia analyses showed that both complexes had impressive SAR values, ranging from 36.83 − 85.81 W/g and 97.67 − 125.58 W/g at therapeutic temperatures of 48 °C and 43 °C, respectively. These results suggest that both complexes may be effective in the treatment of breast cancer using a dual approach, combining the effects of hyperthermia and chemotherapy (Gajare et al. 2022). In another study, CuO NPs (25, 50, and 100 μg/ml), hyperthermia (41 °C for 1 h), and irradiation (200 cGy) were able to inhibit the proliferation of MCF7 cells and induce cell apoptosis via MMP collapse (Ghaleh et al. 2019).

**Fig. 10:**
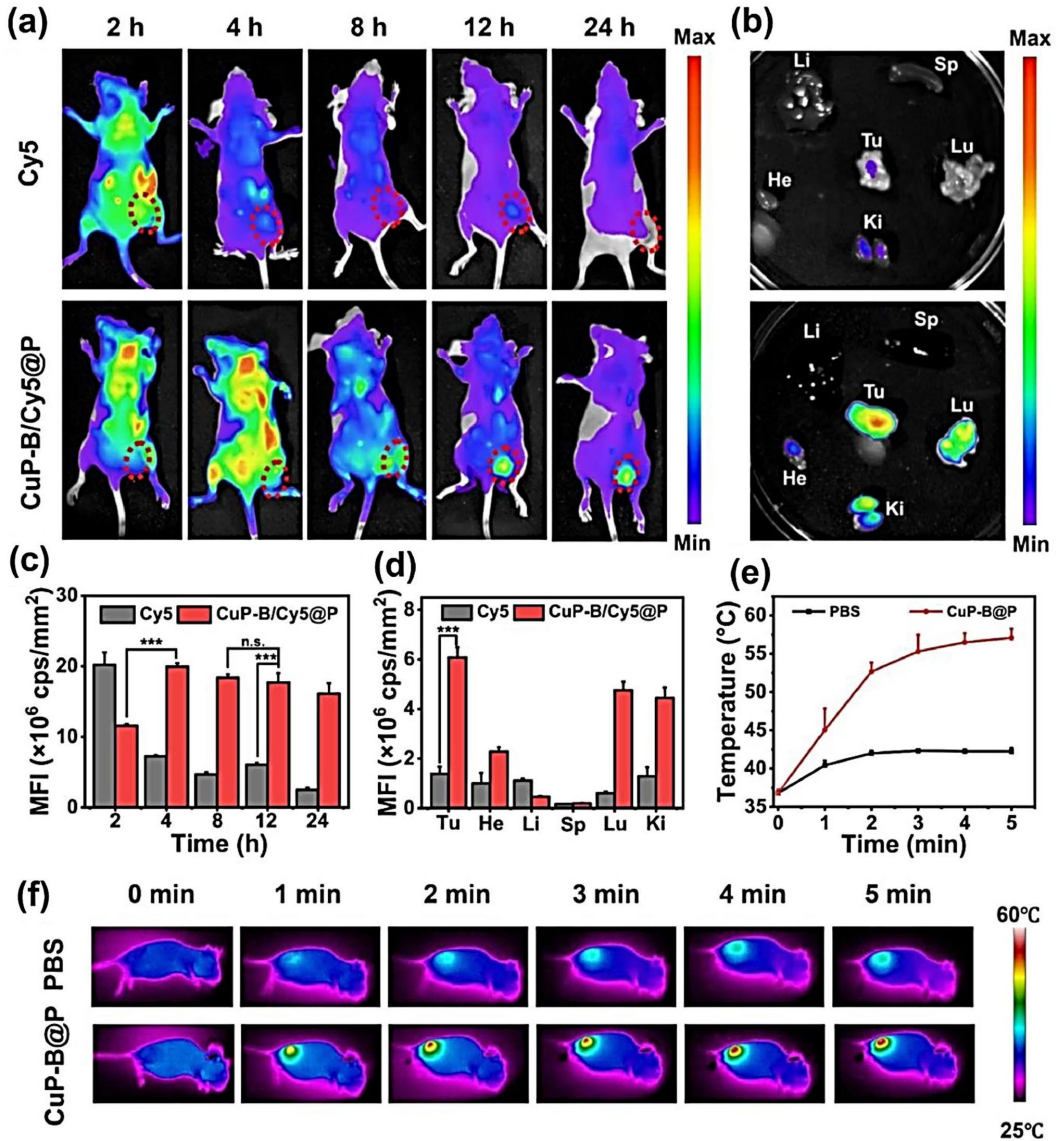
**a** In vivo fluorescence imaging of mice bearing MCF-7 tumor over time after intravenous injection of free Cy5 or CuP-B/Cy5@P. **c** Corresponding quantitative analysis of fluorescence intensity in the tumor region. **b** Ex vivo fluorescence imaging of tumors and major organs, together with **d** Corresponding quantitative analysis. **e** and **f** Infrared thermal imaging of mice bearing MCF-7 tumors and the temperature at the tumor sites after intravenous injection of PBS or CuP-B@P during 808 nm laser irradiation (1 W/cm.^2^)

In 2023, Huang et al. reported Cu-doped polypyrrole for cancer treatment via hyperthermia-triggered nitric oxide (NO) release. According to studies, NO as a gaseous medium has a significant role in tumor progression. Thus, high concentrations of NO can lead to mitochondrial dysfunction and DNA damage. The delivery and release of NO in gas therapy to eradicate malignant tumors at non-toxic levels is challenging and unpredictable. To overcome these challenges, a multifunctional nanocatalyst called Cu-doped polypyrrole (CuP) was developed as a smart nanoplatform (CuP-B@P). This nano-platform is designed to target the NO precursor BNN6 to tumors and release NO. In the abnormal metabolic conditions of the tumor, CuP-B@P catalyzes the conversion of the antioxidant GSH to GSSG and excess H_2_O_2_ to ^−^OH through the copper(I)/copper(II) cycle. This process leads to oxidative damage to tumor cells and the release of BNN6. Moreover, when irradiated with laser light, the nanocatalyst CuP can absorb photons and convert them into hyperthermia, which further enhances the catalytic efficiency and pyrolyzes BNN6 into NO. Through the synergistic effects of hyperthermia, oxidative damage, and NO release, the nanocatalyst achieves nearly complete eradication of tumors within the body while causing minimal harm or toxicity. In this study, the distribution of CuP-B@P was investigated in vivo after intravenous injection. The fluorescence of CuP-B/Cy5@P at tumor sites elevated from 2 to 4 h and remained high from 4 to 24 h (Fig. 10a and c). On the other hand, the fluorescence of free Cy5 initially maintained a robust presence throughout the entire body but rapidly decreased, indicating that the tumor was not targeted. After the experiment, the major organs and tumors were examined using an in vivo imaging system. The analysis showed that the fluorescence intensity in the distant tumor tissue was significantly stronger in the CuP-B/Cy5@P group than in the Cy5 group, confirming the specific accumulation and storage of CuP-B/Cy5@P in the tumor (Fig. 10b and d). Next, in vivo photothermal imaging of CuP-B@P was performed using a thermal imager. When the tumor tissue in the CuP-B@P group was exposed to laser irradiation of 808 nm (1 W/cm^2^, 5 min), the temperature gradually increased and reached 57 °C after 5 min. This temperature was significantly higher than the temperature (40 °C) observed in the PBS group (Fig. 10e and f) (Huang et al. 2023).

**Fig. 11:**
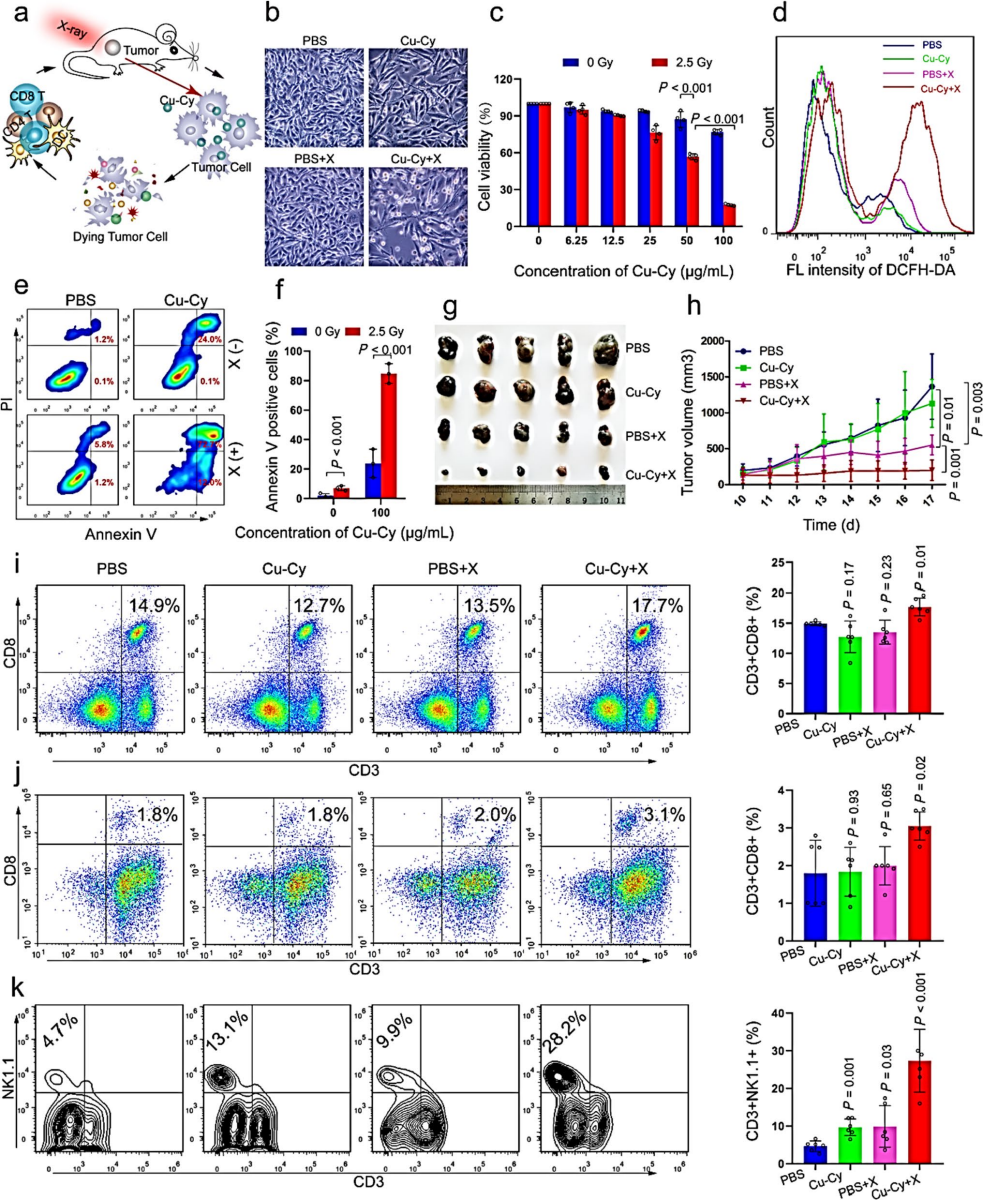
**a** Schematic representation of the use of Cu-Cy NPs for simultaneous radiotherapy, oxidative therapy, and immunotherapy in the treatment of melanoma. **b**) Morphological images of B16 cells treated with Cu-Cy NPs (~ 40 nm) under X-ray irradiation (0 or 2.5 Gy) **c** Cell viability of B16 cells treated with different concentrations of Cu-Cy NPs under 2.5 Gy X-ray irradiation compared to other groups. **d** Increased intracellular ROS levels in B16 cells treated with Cu-Cy NPs and X-rays compared to other groups. **e**, **f** The highest rates of cell apoptosis and/or necrosis in B16 cells treated with Cu-Cy NPs and X-rays compared to other groups. **g** Tumor volumes in different groups at the end of treatment. **h** Tumor growth curves in different groups. **i**–**k** Tumor tissue using flow cytometry to detect changes in infiltrating immune cells. **i** Percentage of CD8 + T cells in the spleen of mice treated with Cu-Cy NPs and X-rays compared to other groups. *P* value versus the PBS group (*n* = 6). **j**, **k** Percentage of CD8 + T cells and NK cells in tumor tissue of mice treated with Cu-Cy NPs and X-rays compared to other groups. *P* value versus the PBS group

According to various studies, copper-based compounds can enhance the effectiveness of immunotherapy in the treatment of cancer (Guo et al. 2022; Zhao et al. 2018; Kong and Sun 2023). Unlike traditional cancer treatments, such as chemotherapy and radiotherapy, which target both cancer and healthy cells, immunotherapy focuses on strengthening the body's immune system to specifically recognize and eliminate cancer cells. One possible way in which copper can improve immunotherapy is by releasing DAMPs from dying cancer cells. DAMPs are molecules that can trigger the immune system to initiate a response against tumors. Treatment with copper can also increase the activity of immune cells, such as T cells and natural killer cells, which have a main effect in the recognition and elimination of cancer cells (Hu et al. 2020). Zhang et al. conducted a study where they explored the application of nanoparticles containing copper and cysteamine to deliver radiation therapy, oxidative therapy, and immunotherapy simultaneously to treat melanoma (Fig. 11a) (Zhang et al. 2020). This research highlights the potential of metal complexes, particularly copper-based compounds, to enhance the outcomes of immunotherapy in cancer treatment. The distribution of Cu-Cy nanoparticles (NPs) within cells was investigated by confocal fluorescence microscopy. The results showed that the uptake of Cu-Cy NPs into the nucleus increased significantly after 6 h of incubation compared with 2 and 4 h. The cytotoxicity of Cu-Cy on B16 cells was evaluated using the CCK8 viability assay. After cells were incubated with various concentrations of Cu-Cy for 24 h, they were irradiated with either 0 or 2.5 Gy of X-rays. The results shown in Fig. 11b and c confirm that the viability of the cells in the control group (Cu-Cy only) did not decrease noticeably. However, in the 2.5 Gy group, there was a dose-dependent decrease in cell viability, indicating that the Cu-Cy NPs have weak toxicity to cells but can be activated by X-rays to cause significant cytotoxic activity. In Fig. 11d, the Cu-Cy + X-ray group showed higher green fluorescence of dichlorofluorescein (DCF) compared with the PBS, Cu-Cy, and PBS + X-ray groups, demonstrating the production of ROS. In the apoptosis assay, the Cu-Cy group exhibited a low apoptosis rate of 24.1%, whereas the combined treatment with Cu-Cy and X-ray showed a significant killing effect with an apoptosis rate of about 84.7% (Fig. 11e and f). In the in vivo study, the PBS and Cu-Cy groups had minimal effects on tumor proliferation, whereas the PBS + X-ray and Cu-Cy + X-ray groups illustrated a strong antiproliferative effect from day 12. The Cu-Cy + X-ray group had the most significant inhibitory effect (Fig. 11g and h). No noticeable changes in body weight or pathological damage to the spleen were observed in any of the groups. These results indicate that the cytotoxicity of Cu-Cy nanoparticles can be enhanced by X-ray irradiation. Cu-Cy-based PDT generates ROS that leads to tumor cell destruction and induction of an immune response. The immune response in the tumor and spleen was examined and it was concluded that only Cu-Cy + x-ray treatment increased CD4 + T and CD8 + T cell levels in the spleen (Fig. 11i). However, no significant changes were observed in other immune cells in the spleen. To understand the inhibitory potency of Cu-Cy-based PDT, the infiltration of immune cells into the TME was investigated. Tumor tissues treated with Cu-Cy + X beams had a higher proportion of dendritic cells (DCs), cluster of differentiation 8 with T cells (CD8 + T cells), and Natural Killer (NK) cells (Fig. 11j and k). Mature DCs play a critical role in initiating an effective adaptive immune response, and activated NK cells can support mature DCs and kill tumor cells directly. Cu-Cy-mediated PDT also reduced the number of M2 macrophages in the TME, whereas the proportion of M1 and MDSC macrophages did not change significantly. In summary, Cu-Cy-based PDT elicits an immune response by increasing CD4 + T and CD8 + T cell numbers in the spleen and promoting infiltration of DCs, CD8 + T, and NK cells into the TME. This immune response, together with the reduction of M2 macrophages, contributes to the cytotoxicity of Cu-Cy-based PDT (Zhang et al. 2020).

In general, copper nanoparticles are promising in cancer immunotherapy because they enhance immune-based therapies (Xu et al. 2023). They can be used as adjuvants in cancer vaccines to enhance immune response and attack cancer cells. Copper nanoparticles can also be combined with immune checkpoint inhibitors to target them to the tumor, increasing the therapeutic efficacy of immunosuppressive factors that promote a more favorable immune response against cancer cells. Although research is still at an early stage, copper nanoparticles can enhance the effectiveness of immunotherapeutic approaches, which requires further studies to optimize (Wang et al. 2019a, b; Li et al. 2023). Generally, the reports on copper-based nanoparticles are also limited to in vitro, in vivo*,* or ex vivo phases (Kang et al. 2023). The clinical implementation of these nanoparticles remains a challenge, and more efforts should be directed to in vivo and ex vivo studies [196]. Compared to other metallic nanoparticles (such as silver, gold, or platinum) copper nanoparticles have not been extensively studied in biomedical applications. Although the number of scientific reports on copper nanomaterials is increasing, more research is needed before they can be used in clinical trials (Gu et al. 2023; Ilbasmis-Tamer et al. 2023; Abu-Serie and Abdelfattah 2023; Zhuang et al. 2022; Wózniak-Budych et al. 2023). Some examples of copper-based compounds and nanoparticles with different applications and mechanisms of action are listed in Table 1.

**Table 1:** Copper-based compounds and nanoparticles with various applications and mechanisms of action

	Copper-based compounds	Cancer cell line	Function description	Mechanisms of action
Therapeutic agent	Copper(II) complex containing pyridine-2,6-dicarboxylate (Abdolmaleki et al. 2023b, a)			
		• BEL-7404	• Binding to DNA • Cell cycle arrest • ROS generation • MMP collapse	• Apoptosis
	Copper(II) complexes containing 2-(2′-pyridyl)benzimidazole and/or 5-chloro-2-(2′-pyridyl)benzimidazole (Cai et al. 2021)	• A549 • HeLa • SGC-7901		
	Copper(II) complex containing DSF (Xu et al. 2020)	• MM		
	Trinuclear copper(II) complex containing thiophene-2-formaldehyde TSC (Wang et al. 2024)	• T24	• Prevention of migration and invasion of vascular endothelial cells • Cell cycle arrest • Induction of autophagy	• Apoptosis • Antiangiogenesis
	Phen-copper(II) complex containing a triphenylphosphonium group (Zheng et al. 2023)	• TNB • MDA-MB-231	• Induction of mitophagy • Mitochondrial damage • Reduction of the ability of HUVEC to form tubes • Decrease in expression of VEGF and CD34 in HUVEC • Inhibition of vasculogenic mimicry formation	• Antiangiogenesis
	Copper(II) complexes containing 1-adamantoylhydrazone (Rodić et al. 2016)	• Vascular endothelial cells	• Less formation of tube-like structures in EA.hy926 cells	
	ES-Cu (Yang et al. 2023a, b, c, d)	• Colorectal cancer cells	• Aggregation of lipoylated mitochondrial enzymes • Loss of Fe-S Proteins	• Cuproptosis
	HK–Cu (Chen et al. 2017)	• A549 • K562	• Accumulation of ubiquitinated proteins in cells • Strong inhibition of DUBs	• Paraptosis
Diagnostic tool	[^64^Cu]Cu-ATSM (Savi et al. 2017; Nam et al. 2022)	• NCT03951337 • NCT04875871	• Penetration into mitochondria • Reduction from copper(II) to copper(I) • Long-term storage of the metal or radiometal in hypoxic cells	• Contrast Agent in PET/MRI scanning • Therapeutic agent
	^64^CuCl_2_ (Guerreiro et al. 2018)	• PCa • GBM	• High uptake • DNA damage • Genomic instability	• Contrast agent in PET/CT imaging • Therapeutic agent
	Cu(II)-GTSM (Xie et al. 2017)	• AD	• High uptake in brain and spinal cord	• Contrast agent in PET imaging
Combined approach Combined approach	CFp NPs (Koo et al. 2022)	• HeLa	• Release of H_2_O_2_, copper ions and iron ions • Production of O_2_ • Alleviate hypoxia or monitor tumors in vivo	• CDT
	Cu@cLAs (Cui et al. 2023)	• MCF7	• Release of copper(I) and (II) ions that kill cancer cells by delaying the loss of copper metabolism and increasing ROS levels	• CDT
	Cu(ii)Chl-HA NPs (Guo et al. 2019)	• CD44-overexpressing	• Replace magnesium(II) in chlorophyll to increase ^1^O_2_ production • Achieve receptor-mediated targeting in tested cells	• PDT
	Ce6-DNAzyme/[Cu(tz)] (Liu et al. 2021a, b, c)	• MCF7	• Release of a tumor-targeted DNAzyme • Generation of ^1^O_2_ by type II PDT • Trigger a type I response	• PDT
	(GOx@[Cu(tz)]) (Xu et al. 2022)	• Bladder cancer	• Degradation of glucose and GSH • Sensitization of tumor cells to GOx@[Cu(tz)]-associated cuproptosis	• Cuproptosis • PDT
	Cu-Cy (Chen et al. 2022)	• VX2	• Downregulation of PCNA • E-cadherin expression	• PDT
	CCPCA NPs (He et al. 2022)	• A375 • 4T1 • U87MG	• Reduction of hypoxia of the cancer • Release of O_2_ generated by catalase	• PDT
	CuS NPs (Li et al. 2018)	• HeLa	• Direct targeting of cancer cells • Induction of apoptosis through NIR irradiation	• Apoptosis • PTT
	CuS-RNP/DOX@PEI (Chen et al. 2021)	• A375 • MCF7 • Hela	• Release of Cas9 RNP and DOX • Acceleration of the release of therapeutic agents	• Gene therapy • Chemotherapy • PTT
	CuS-RNP@PEI (Yan et al. 2021)	• Malignant neoplasm	• Accumulation of intratumorally infiltrating CD8 T lymphocytes in tumor-bearing mice • Up-regulation of IFN-γ and TNF-α expression levels in tumor tissue • Sensitization of tumors for immunotherapy	• Immunotherapy • PTT
	[Nano-magnetite-Lys@Cu(PPh_3_)I] and [Nano-magnetite-Arg@Cu(PPh_3_)I] (Gajare et al. 2022)	• MCF7	• Impressive SAR values at therapeutic temperatures	• Hyperthermia • Chemotherapy
	CuO NPs (Ghaleh et al. 2019)	• MCF7	• MMP breakdown	• Apoptosis • Hyperthermia
	CuP-B@P (Huang et al. 2023)	• MCF7	• Releases NO • Conversion of GSH to GSSG and H_2_O_2_ to ^–^OH • Oxidative damage Release of BNN6	• Hyperthermia
	Cu-Cy NPs (Zhang et al. 2020)	• B16	• Induction of an immune response • ROS production	• Immunotherapy • PDT • Apoptosis

## Limitations and challenges in the use of copper-based compounds

Although copper-based compounds are promising for cancer therapy, there are some problems with their use. Copper is a necessary trace mineral for the human body, but high concentrations of copper can be toxic (Tang et al. 2023). When using copper compounds in cancer therapy, a balance must be struck between therapeutic efficacy and toxicity to healthy cells and tissues (Wang et al. 2023a, b).

According to studies, increased values of copper have been detected in various types of tumors, which can be attributed to several factors. Tumors with high metabolic demands, especially fast-growing tumors, require copper as a cofactor for enzymes involved in cellular energy metabolism and antioxidant defense. This increased need for copper in cancer cells contributes to the higher copper content (Gao and Zhang 2023; Fiadjoe et al. 2023; Su et al. 2022). In addition, hypoxia, which is common in tumors, leads to upregulation of CTR1. Hypoxia-inducible factor 1-alpha indirectly activates genes involved in copper metabolism, including those that control CTR1, further increasing copper levels in tumor cells. Up-regulation of CTR1 has been observed in a variety of tumors (Lopez et al. 2019; Wooton-Kee et al. 2020). Tumors often exhibit an imbalance in copper levels that affects mitochondrial respiration, glycolysis, insulin resistance, and lipid metabolism. Liver cancer occurs more frequently in patients with Wilson's disease and animal models. There is evidence that the abnormal accumulation of copper may contribute to the transformation of liver cells, although the exact mechanisms are not yet fully understood. Extensive research has shown that cancer cells require an increased copper content to support their rapid growth compared to normal cells (Wooton-Kee et al. 2020). Some studies proved that copper metabolism is closely associated with tumor development. Cancer cells have a higher requirement for copper compared to normal cells, as it is essential for their rapid proliferation and energy needs (Atakul et al. 2020). Copper acts as a cofactor for cytochrome c oxidase, an enzyme involved in ATP synthesis and electron transport in the mitochondrial respiratory chain (Fang et al. 2019). Abnormal copper levels have been detected in the blood and tissue of various cancer patients. Impaired copper homeostasis, as evidenced by notable alterations in copper-binding proteins, is closely associated with cancer occurrence, development, and metastasis (Tsang et al. 2020). To gain more copper, cancer cells often show increased expression of the CTR1. Despite the increased glycolysis observed in cancer cells, the administration of copper chelators can greatly reduce ATP generation. This suggests that tumor cells rely heavily on mitochondrial respiration and oxidative phosphorylation to meet their energy needs. Here, copper may act as a limiting factor in tumorigenesis and cancer progression as it regulates ATP production via oxidative phosphorylation, which is necessary to meet the high energy demands of rapidly proliferating cancer cells (Atakul et al.^2020^; Fang et al. 2019; Tsang et al. 2020).

Copper is an important cofactor for the autophagy kinases ULK1 and ULK2 and can increase the activity of these kinases, leading to higher autophagy flux. This promotes the proliferation and viability of neoplastic lung cells. In Wilson's disease, copper accumulation in the liver is associated with autophagy activation. In colorectal cancer with KRAS gene mutations, tumor cells take up more copper through macro-pinocytosis, which promotes tumor growth. On the other hand, lower availability of copper suppresses the growth of KRAS-mutated cancer cells (Tsang et al. 2020; Aubert et al. 2020). Research suggests that enhanced copper levels can stimulate tumor progression by boosting mitochondrial energy metabolism, leading to the mis-folding of tumor-associated proteins, triggering the activation of signaling pathways related to tumor development, and influencing the activity of autophagy kinases (Tsang et al. 2020; Aubert et al. 2020). Copper has a significant impact on the promotion of angiogenesis, as demonstrated in the 1979 study by McAuslan et al. Subsequent research has shown that copper stimulates endothelial cell migration, a crucial step in the formation of blood vessels. Excessive copper levels are observed during the process of neovascularization, and the addition of copper promotes the growth of blood vessels. In animal models, inhibition of copper leads to a decrease in blood vessel formation. Copper promotes angiogenesis by several mechanisms, including direct binding to growth factors involved in angiogenesis, increasing nitric oxide production, regulating angiogenic factors, such as FGF and IL-1α, and modulating the activity of the NF-κB cells. Copper also interacts with HIF-1α, an important regulator of angiogenesis, influencing its transcriptional activity and promoting tumor growth (Karginova et al. 2019). Clinical trials have shown a remarkable relationship between serum copper levels and HIF-1α levels in liver cancer patients, demonstrating that copper has a considerable role in HIF-1α activation and liver cancer progression. Inhibition of copper chaperone proteins can disrupt copper balance, inhibit tumor angiogenesis, and induce apoptosis in breast cancer cells. The results also suggest that copper has a significant effect on tumor angiogenesis (Wu et al. 2019). Copper has a remarkable role in advancing the growth of blood vessels in tumors by influencing the activity of HIF-1α and regulating the release of various factors that promote blood vessel formation. By reducing the copper content in tumor tissue or preventing copper transport in cancer cells, it may be possible to effectively block the growth of blood vessels in tumors. This could be a valuable therapeutic strategy for the treatment of cancer (Karginova et al. 2019; Wu et al. 2019).

Copper plays a decisive role in tumor cells, influencing their growth, invasion, and spread. It influences these processes through the activity of LOX enzymes, which contribute to the strengthening of the extracellular matrix. LOX and LOXL proteins depend on copper for their function, and the copper exporter ATP7A protein transports copper for them. In breast cancer, inhibition of ATP7A disrupts LOX activity and impedes metastasis. High levels of LOXL2 are associated with aggressive breast cancer by reducing cell adhesion and promoting EMT. Targeted inhibition of LOX and LOXL by copper can successfully inhibit the invasion and spread of cancer cells. The MEMO1, another copper-dependent protein, is involved in cell movement and metastasis of breast cancer (Shanbhag et al. 2019). Treatment with a copper chelator called TTM lowers copper levels, inhibits MEMO1, delays blood vessel formation, and prevents breast cancer metastasis. Lowering copper levels by chelation or targeting copper transporters not only suppresses the growth of cancer cells but also downregulates the expression of genes associated with EMT. Copper also plays a role in the activation of genes associated with EMT, as it is involved in the binding of HIF-1α to specific gene sequences (Shanbhag et al. 2019). Copper can affect the activity of LOX and LOXL enzymes, the expression of MEMO1, and the binding of HIF-1α to target genes with HRE (Hypoxia Response Elements) sequences. This regulation of gene expression leads to the modulation of EMT-related genes and the promotion of tumor cell metastasis [216]. Consequently, the use of copper in therapy can interrupt this signaling mechanism and effectively prevent the spread of tumor cells (Shanbhag et al. 2019). Tumor cells undergo a unique metabolic process known as aerobic glycolysis. This allows them to adapt to different oxygen levels, promote cell proliferation, and prevent cell death. Under aerobic conditions, tumor cells increase their glycolysis, glucose uptake, and lactate production while weakening oxidative phosphorylation. This metabolic reprogramming provides tumor cells with the energy and nutrients they need to survive and proliferate, giving them an advantage over normal cells. Hypoxia, or low oxygen levels, has a vital role in stimulating the overexpression of genes related to glycolysis in tumor cells (Wu et al. 2019; Shanbhag et al. 2019; Feng et al. 2009). The HIF-1α protein is an imperative regulator of this process and is involved in cancer development. Copper also affects the regulation of glycolysis and the activity of HIF-1α. It can prevent the degradation of HIF-1α and thus improve its function by interfering with the activation of certain proteins. However, copper is also crucial for the functioning of respiratory complex IV in mitochondria, which affects overall cellular activity. Copper chelators can decrease mitochondrial respiration and activate glycolysis (Wu et al. 2019). Therefore, the involvement of copper in metabolic reprogramming of tumors is not a simple correlation, but rather a complex interplay of different pathways. To develop better treatment approaches, more in-depth investigation is needed to fully understand the underlying mechanisms of copper's role in the metabolic reprogramming of tumors (Wu et al. 2019; Feng et al. 2009; Jensen et al. 2019).

## Conclusions

Copper complexes synthesized from various organic ligands, such as thiosemicarbazone, Schiff base, imidazole, benzimidazole, pyrazole, triazole, pyridine, and isatin diamine, as well as 1,10-phenanthroline derivatives, have demonstrated their potential as anticancer agents. They can induce cell death, and inhibit angiogenesis, tumor growth and metastasis via various mechanisms. However, side effects must be considered, such as potential toxicity, lack of selectivity, and the development of drug resistance. Clinical studies with copper complexes, particularly [^64^Cu]Cu-ATSM, have shown promise for imaging tumor hypoxia and predicting patient response to therapy. Ongoing research and clinical studies are investigating the use of copper complexes in various medical fields. Copper nanoparticles hold promise for cancer therapy due to their unique properties, as they can selectively target cancer cells and deliver drugs. Copper nanoparticles have shown remarkable efficacy in CDT due to their properties that can generate ROS and induce tumor cell death. They can be used as effective photosensitizers to selectively target and destroy cancer cells by light activation in phototherapy. The use of copper nanoparticles in hyperthermia promises to improve the effectiveness of cancer treatment. Copper nanoparticles can be used to generate heat when exposed to certain wavelengths of light or alternating magnetic fields. This ability to generate heat makes them suitable for targeted heating of tumor cells. By targeting copper nanoparticles in the tumor and activating them with external stimuli, it is possible to raise the temperature of the cancer tissue while causing as little damage as possible to the healthy surrounding cells. Copper nanoparticles can be used for cancer immunotherapy because they support immune-based treatments. When used as an adjuvant in cancer vaccines, they can improve the immune response and attack cancer cells. In combination with immune checkpoint inhibitors, copper nanoparticles can also be targeted to tumors to improve the effectiveness of therapy. While copper is an essential mineral needed for various bodily functions, excessive amounts can be harmful, such that high levels of copper in the body may be associated with an enhanced risk of certain cancers. Copper is involved in the formation of free radicals, unstable molecules that can damage DNA and other cellular components. This oxidative damage can lead to mutations and the development of cancer cells. In addition, copper is also involved in angiogenesis, the process by which new blood vessels are formed. Tumor cells rely on angiogenesis to grow and spread, and elevated copper levels can promote this process. Lowering copper concentrations or inhibiting copper transport in tumor cells may be promising strategies to inhibit angiogenesis and metastasis of tumors. However, it should be noted that the correlation between copper and cancer is complex, and further study is needed to fully understand the mechanisms involved.

## Data Availability

No datasets were generated or analysed during the current study.

## Abbreviation

AO/EB: Acridine orange/ethidium bromide
ATP7A: ATPase copper transporting alpha
ATSM: Diacetyl-bis(N4-methylthiosemicarbazone
BNN6: N,N′-di-sec-butyl-N,N′-dinitroso-1,4-phenylenediamine
CAM: Chick embryo chorioallantoic membrane
COX2: Cyclooxygenase-2
Cu,Zn-SOD: Copper, zinc superoxide dismutases
DAMPs: Damage-associated molecular patterns
DLAT: Dihydrolipoamide S-acetyltransferase
DLST: Dihydrolipoamide S-succinyltransferase
Dpq: Dipyrido-[3,2-d:2’,3’-f]-quinoxaline
EPR: Enhanced permeability and retention
EMT: Epithelial-to-mesenchymal transition
FAZA: Fluoroazomycin arabinoside
FDG: Fluorodeoxyglucose
FMISO: Fluoromisonidazole
FGF: Fibroblast growth factor
GTSM: Glyoxal-bis(4-methylthiosemicarbazonato)
HIF-1α: Hypoxia-inducible factor 1-alpha
HSA: Human serum albumin
Htdp: 2-[(2-(2-Hydroxyethylamino)ethylimino)methyl]phenol
IL-1α: Interleukin-1 alpha
KRAS: Ki-ras2 Kirsten rat sarcoma viral oncogene homolog
LOX: Lysyl oxidase
LOXL: Lysyl oxidase-like
MAMs: Mitochondria-associated membranes
MCU: Mitochondrial uniporter
MEMO1: Mediator of ErbB2-driven cell motility
MELAS: Mitochondrial encephalopathy, lactic acidosis, and stroke-like episodes
NF-κB: Nuclear factor kappa B
Nrf2: Nuclear factor erythroid 2-related factor 2
PDGFB: Platelet-derived growth factor B
PDGFRbeta: Platelet-derived growth factor receptor beta
RNA: Ribonucleic acid
SDHB: Succinate dehydrogenase complex iron sulfur subunit B
Tie2: Immunoglobulin-like loops and epidermal growth factor homology domains-2
Tmp: 3,4,7,8-Tetramethyl-1,10-phenanthroline
